# Efficient oxidative degradation of Azo dyes by cobalt(II) porphyrin complex supported on modified bentonite and chitosan: structural characterization and mechanistic insight

**DOI:** 10.1038/s41598-025-19788-9

**Published:** 2025-10-01

**Authors:** Sahar H. El-Khalafy, Mahmoud T. Hassanein, Nehal A. Salahuddin, Mohamed M. Alaskary

**Affiliations:** Department of Chemistry, Faculty of Science, University of Tanta, Tanta, 31527 Egypt

**Keywords:** Oxidative degradation, Metalloporphyrin, Bentonite clay, Chitosan, Hydrogen peroxide, Methyl orange dye, Methyl red dye, Orange II dye, Chemistry, Materials science

## Abstract

Cobalt(II) complex of 5, 10, 15, 20 tetrakis [4-(hydroxy)phenyl] porphyrin [Co(II) TP-OHPP] **1**, [Co(II) TP-OHPP]/CPTES−Bentonite Clay **2** composite, [Co(II)TP-OHPP]**/**CPTES−Bentonite Clay/ Chitosan **3** composite, has been used to study the catalytic degradation of a group of azo dyes (e.g., Methyl Orange, Methyl Red, and Orange II) by hydrogen peroxide in an aqueous solution. ^1^HNMR, FT-IR, SEM, XRD, TGA, DLS and UV-Vis were used to characterize the structures and characteristics of the soluble and supported catalyst. The UV-Vis spectra of the reaction mixture were recorded at λmax = 464, 505, and 485 nm for M.O, M.R, and O II dyes, respectively, in order to track the oxidation reaction. [Co(II)TP-OHPP]/CPTES−Bent/Cs **3** composite, shows the highest supported catalyst in the degradation process of the three anionic dyes with superior catalytic efficiency, underscoring its enhanced degrading capabilities. A comprehensive study was conducted using advanced techniques to evaluate its performance. Total organic carbon analysis (TOC) showed oxidation of M.O, M.R, and O II dyes revealing the elimination of 69%, 85%, and 66% mineralization under mild reaction conditions. The composite stability and reusability were evaluated. GC-MS analyses were used to identify any remaining organic components in the reaction mixture. There has been discussion about the oxidation reaction’s mechanism.

**Supplementary Information:**

The online version contains supplementary material available at 10.1038/s41598-025-19788-9.

## Introduction

The chemical industry, particularly in textile manufacturing and dyeing, releases harmful pollutants like complex chemical compounds known as azo dyes have an azo bond (−N = N−) and one or more auxochromes and chromophores in their molecular structure (e.g., Methyl Orange[(E)−4-((4-(dimethylamino)phenyl)diazenyl)benzene-sulfonate], Methyl Red [2-((4-(dimethylamino)phenyl)diazenyl)benzoic acid], and Orange II[sodium 4-((2-hydroxynaphthalen-1-yl)diazenyl)benzenesulfonate])^1–3^, which are toxic, non-biodegradable, and resistant to degradation, leading to long-term water pollution, oxygen depletion, and potential carcinogenic byproducts^4,5^. Conventional treatments are ineffective, necessitating advanced methods like advanced oxidation, biological treatment, and electrochemical processes to convert these dyes into non-toxic, biodegradable substances and reduce their environmental impact^6–15^.

Numerous physical, biological, and chemical treatment methods have been studied and applied to remove these organic pollutants from water. Advances in organics removal from wastewaters have resulted in the creation of advanced oxidation processes (AOPs), which are essentially based on generating highly reactive species such as hydroxyl radicals (HO^•^) and per-hydroxyl radicals (HO_2_^•^)^16^. Fenton’s reagent oxidation has emerged as one of the most appealing and promising treatment techniques for the efficient breakdown of dyes and the elimination of numerous dangerous organic contaminants^17–20^.

Homogeneous metalloporphyrin’s are most effective catalysts for different oxidation reactions^21–25^, however homogeneous catalysis shows some limitations associated with their self-decomposition, including dimerization, recovery, and reuse. Thus, the immobilization of these porphyrins into solid support such as clay, zeolites, hydrogels, microparticles, chitosan, and other supporting materials have been reported as an effective strategy to increase their stability and applicability^26–38^. Herein, a chitosan and Bentonite clay were used as polymer matrix. Chitosan a deacetylated derivative of chitin characterized by high hydrophilicity and many hydroxyls and amino groups. Chitosan widely applied as supporting material due to it is environmentally friendly biodegradable and biocompatible material. However, CS has several disadvantages, such as poor mechanical strength, easy agglomeration, and its solubility in dilute acids. Immobilization of chitosan in a low-cost material such as sand, montmorillonite and bentonite clay have been applied to eliminate these short comings^39^. Bentonite’s high surface area and surface energy give it a potent adsorption capacity. However, a layer of water molecules covers its inside surface due to its surface negative charge and abundance of exchangeable positive ions. Because of this interior layer of water molecules, bentonite is a powerful hydrophilic substance. Therefore, bentonite is not suitable for the adsorption of organic contaminants. Clay-based nanocomposites and bio-nanocomposites are employed in numerous fields because of their functional characteristics and nanoscale architectures^40^. Clays can be combined with chitosan, which is thought to be a suitable structure for clay modification, because of their highly reactive surfaces and expandable surface areas^41^. This is seen to be a promising approach to creating these hybrid materials, which show even better behavior that represents the inherent qualities of both constituents^40,42^.

**Fig. 1:**
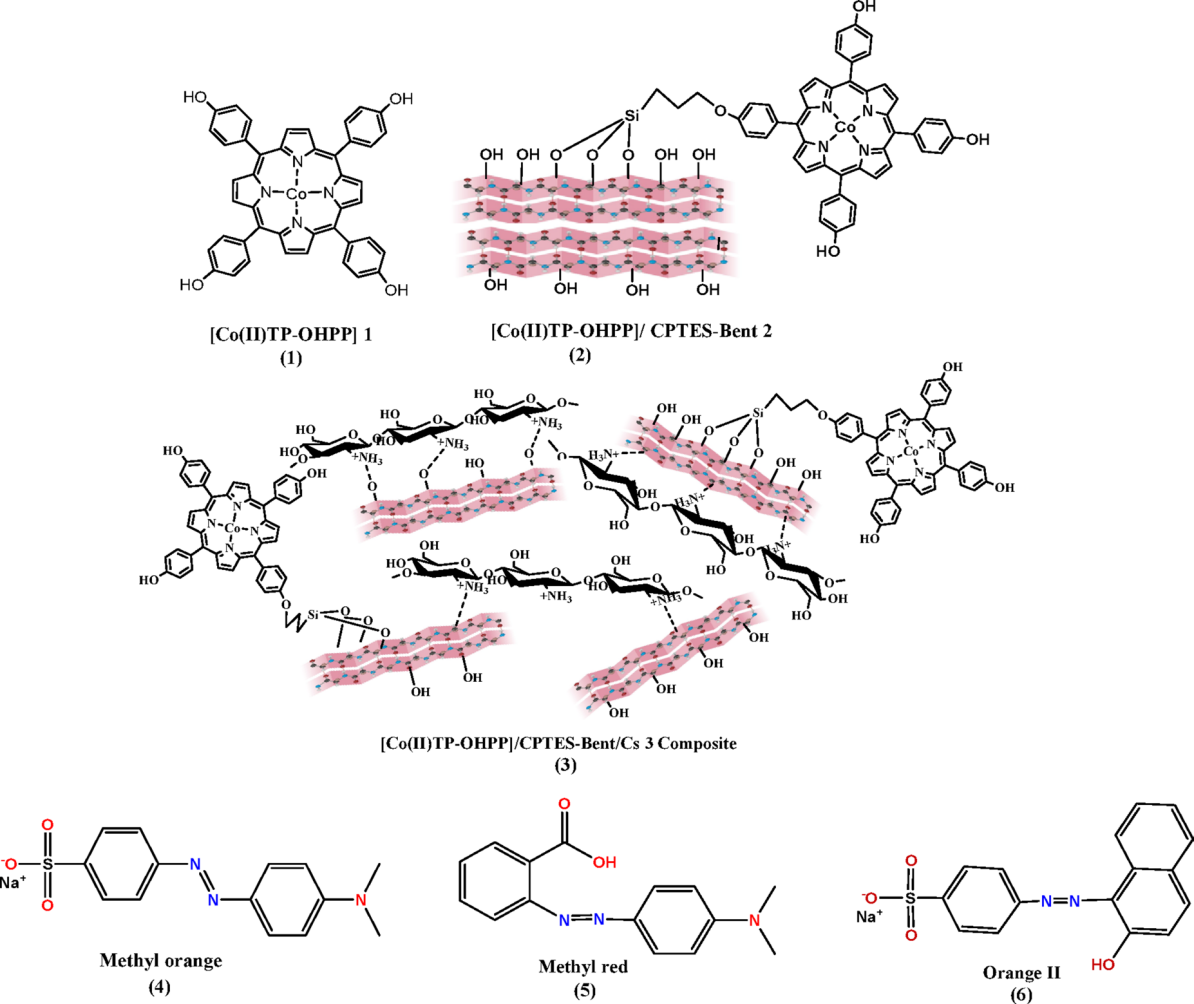
(1) [Co(II)TP-OHPP] 1, (2) [Co(II)TP-OHPP]/CPTES-Bent 2, (3) [Co(II)TP-OHPP]/CPTES−Bent/Cs 3 composite, (4) Methyl Orange, (5) Methyl Red, (6) Orange II.

In this study, the use of 5,10,15,20-Tetrakis(4-hydroxy phenyl) porphyrinato] cobalt (II) complex [Co(II) TP-OHPP] in homogeneous catalysis for catalytic degradation of methyl orange (M.O), methyl red (M.R), and orange II (O II) in an aqueous solution with hydrogen peroxide exhibits high catalytic activity with certain limitations, as previously discussed. According to these characteristics, here we found that bentonite clay/chitosan modified by (3-chloropropyl) tri-ethoxy silane CPTES/Bent/Cs act as efficient support to the catalyst [Co(II) TP-OHPP], enhancing its catalytic activity, stability, and sufficiently fast in the oxidative degradation reaction of the reported anionic azo dyes Figure (1).

## Experimental framework

### Chemicals and reagents

Sigma-Aldrich provided the (3-Chloropropyl)triethoxysilane (95%),p-hydroxybenzene carbaldehyde (98%), propionic acid (99.5%), Methyl orange, Methyl Red, Orange II dyes (dye content ≥ 85%), chitosan network (Mwt 150,000 g/mol), and cobalt (II) chloride hexahydrate (≥ 97%). Merck Darmstadt, Germany, supplied the pyrrole (97%) was distilled prior to usage, and bentonite clay, which had a surface area of 67.6 m^2^/g and a cation exchange capacity (CEC) of 104 meq/100 g. Triethylamine (99%) and toluene (99.9%) were acquired from (Alpha-Chemika, India). N, N-dimethylformamide (DMF) (99.8%), chloroform (99.4%), methylene chloride (99.9%), anhydrous potassium carbonate, methanol (99.9%) were provided by (El-Nasr Pharmaceutical Chemicals, Egypt). The supplier of silica gel (60–120 mesh) was Fisher Co., in New Jersey, USA.

### Instrumental measurements

The chemical shifts were obtained by recording ^1^HNMR spectra at 400 MHZ using a Bruker Avance II spectrometer in the presence of CDCl_3_. Utilizing a UV-1800 UV-Vis scanning spectrophotometer manufactured by (SHIMADZU in Kyoto, Japan), the UV-visible spectra ranging from 200 to 800 nm were assessed. Using a JASCO FT-IR-4100, Japan, apparatus and chloroform as the solvent, FTIR was performed in the 400–4000 cm^–1^ range with KBr pellets for powdered polymers or fluid specimens of semi-viscous polymers. The crystallographic structure was examined utilizing an Italian-made APD 2000 Pro powder diffractometer for X-rays. The wavelength of the Cu-K radiation employed in the experiment is 1.5406 Å. The scan was performed with an angle range of 5 to 90 and at a rate of 0.05/sec at 45 kV and 0.8 mA. An extensive analysis was performed using the JSM-IT200 In Touch Scope™ SEM. Zetasizer (Malvern Nano Z, UK) was used to conduct the zeta potential measurements. Average particle size was measured using (Malvern Panalytical Mastersizer 3000 Is, UK), which operates based on Dynamic Light Scattering (DLS). TOC measurement using Sievers M5310 C Laboratory TOC Analyzer. Thermo Scientific ISQ single quadrupole gas chromatography-mass spectrometry (GC-MS) apparatus was used to perform the GC-MASS analysis.

### Preparation of the homogenous and heterogeneous catalysts

#### Synthesis of 5,10,15,20 Tetrakis(4-hydroxy phenyl) porphyrin [TP-OHPP

Tetra (*p*-hydroxy) phenyl porphyrin [TP-OHPP] was prepared and purified according to the previous method^43^.

^1^HNMR (400 MHz, in DMSO): δ (ppm) − 2.84 (s, 2 H, NH), 7.24 (d, 8 H, ArH), 8.03 (d, 8 H, ArH), 8.9 (s, 8 H, βH), 10.03 (s,4 H, OH).

UV-Vis (CHCl_3_) λ_max_ : 416, 516, 554, 592 and 649 nm.

IR (ν, cm^–1^): 3423 ν(O−H, NࣧH), 1469 ν(C = N), 808, ν(macrocycle ring’s N–H out-of-plane bending vibration), 1599 ν(N−H bending),1228 ν(C−N), 1001 ν(Co−N).

#### Synthesis of [5,10,15,20-Tetrakis(4-hydroxy phenyl) porphyrinato] Cobalt (II) complex [Co(II) TP-OHPP] 1

Co(II) porphyrin complex [Co(II) TP-OHPP] **1** was obtained according to the reported method^44^.

^1^HNMR (400 MHz, in DMSO): δ (ppm) 7.25 (d, 8 H, ArH), 7.9 (d, 8 H, ArH), 9.13 (s, 8 H, βH), 10.03 (s,4 H, OH).

UV-Vis (CHCl_3_) λ_max_ : 448, 544, 581 nm.

IR (ν, cm^–1^): 3423 ν(O−H) become broad and slightly shifted, and new band appears.

around 1001 ν(Co−N).

#### Modification of the bentonite clay by (3-chloropropyl)triethoxysilane (CPTES)

First, CPTES was used to functionalize the bentonite surface. This was accomplished by dispersing 2 g of bentonite in 35 mL of dry toluene while stirring constantly. Next, 3 mL of 3-chloropropyltriethoxysilane (CPTES) was added to the Bent solution drop by drop. The resulting Liquid was then constantly swirled overnight at 110 °C while reflux was in effect. The result, bentonite−Cl, was then filtered, repeatedly washed with 30 mL of toluene, and allowed to dry overnight at 70 °C^45^.

IR (ν, cm^–1^): 3632,3429 ν(O−H), 2954 ν (– CH_2_ –), 1641 ν(H–O–H), 1032 ν(Si–O–Si), 692 ν(C–Cl).

#### Synthesis of [Co(II)TP-OHPP]/CPTES−bentonite clay 2 composite

The [Co(II)TP-OHPP] complex was covalently attached to bentonite−Cl using a previously documented technique^46^, with minor adjustments, (0.01 mmol) of cobalt porphyrin complex in DMF 30 milliliters was added to a suspension of bentonite−Cl (1.0 g) at 80 degrees Celsius for four hours while 0.5 g of anhydrous potassium carbonate was present.

IR (ν, cm^–1^): 3632,3429 ν(O−H), 2954 ν (– CH_2_ –), 1662 ν(C = C), 1469 ν(C = N), 1032 ν(Si–O–Si).

#### Intercalation of chitosan into bentonite clay (bent/Cs)

One documented technique involved utilizing a chitosanium chain to modify the bentonite clay cationically^47^. After minor adjustments, 2 g Cs (2% w/v) acetic acid solution was heated gradually to 45 °C and stirred all night to guarantee total dissolution.

At the same time,1 g of bentonite was swollen in 100 ml water, sonicated, homogenized, and constantly stirred overnight. Next, combine the two solutions and reflux at 80 °C for 8 h. Stir for another 2 days at room temperature. filter the precipitate, wash it with distilled water, and then bake it for an entire night at 50 °C.

IR (ν, cm^–1^): 3410 ν(O−H), 2924 ν(C−H), 1647 ν(C = O), 1577 ν(N−H deformation vibration), 1420 ν(C−N), 1080 ν(Si–O–Si), 619 ν(Si–O–Al).

#### Modification of the bentonite clay/chitosan by (3-chloropropyl)triethoxy silane (CPTES)

The Bent/Cs was first functionalized via CPTES. To do this, 2 g of Bent/Cs was mixed with 35 mL of dry toluene while being constantly stirred. After that, the Bent/Cs solution was gradually supplemented with 3 mL of 3-chloropropyltriethoxysilane (CPTES). The resultant mixture was then continuously stirred overnight at 110 °C while reflux conditions were maintained. The following step involved filtering the product, CPTES− Bend/Cs, washing it twice with 30 mL of toluene, and drying it overnight at 70 °C^45^.

IR (ν, cm^–1^): 3410 ν(O−H), 2936 ν(– CH_2_ –), 1577 ν(N−H), 1420 ν(C−N), 1080 ν(Si–O–Si),780 ν(C–Cl).

#### Synthesis of [Co(II)TP-OHPP]/CPTES−bentonite clay/chitosan 3 composite

CPTES–Bent/Cs (1.0 g) was dispersed in DMF (30 ml). Then, 0.01 mmol of cobalt porphyrin complex and 0.5 g of anhydrous potassium carbonate were added and heated to 80 °C for 4 h. To facilitate the covalent attachment of the [Co(II)TP-OHPP] complex on (CPTES/Bent/Cs) according to a previously published method, with minor modifications^46^.

IR (ν, cm^–1^): broad band at 3426 ν(O−H, N−H), and 2924 ν(C−H), 1442 ν(C = N, C = C), 1074 ν(Si−O).

#### Catalytic degradation of methyl orange, methyl red, and orange II dyes using [Co(II)TP-OHPP] 1, [Co(II)TP-OHPP]/CPTES−bentonite clay 2 composite, [Co(II)TP-OHPP]/CPTES−bentonite clay/chitosan 3 composite by H_2_O_2_

The degradation of the three azo dyes (M.O, M.R, and OII) in aqueous media using [Co(II)TP-OHPP]**1**,[Co(II)TP-OHPP]/CPTES-Bent**2**,[Co(II)TP-OHPP]/CPTES−Bent/Cs **3** composite as catalysts, the reaction initiated after the addition of H_2_O_2_ and these Fenton reactions were performed as follows: (9.489 × 10^−4^mol/ml) of [Co(II)TP-OHPP] **1**, (15 × 10^−3^g/ml) of [Co(II)TP-OHPP]/CPTES-Bent **2**,(15 × 10^−3^g/ml) of [Co(II)TP-OHPP]/CPTES−Bent/Cs **3** composite was put in a flask with 10 mL of (1.33 × 10^−4^ M) M.O dye, (1.11 × 10^−4^ M) M.R dye, (1.42 × 10^−4^ M) O II dye aqueous solution with pH was adjusted to 9.0 by using borax and HCl buffer mixture, and the solution was stirred at 40 °C. The reaction was initiated by adding (8 × 10^−2^M) H_2_O_2_, and the rate of deterioration was monitored using UV-Visible spectroscopy. Three milliliter aliquots were removed from the reaction flask and analyzed at predetermined intervals. The UV of the three dyes were calculated at wavelengths of 464, 505, and 485 nm for M.O, M.R, and O II dyes, respectively. The reaction flask was then filled with the aliquots once more. The following first-order relation was used to suit the destruction results:

1 $$\text{ln(A}\circ/\text{A}_\text{t})=\text{k}_\text{obs}\times\text{t}$$

Where *K*_obs_ (min^−1^) is the observed rate constant determined from the slope of the linear plot of ln(*A*o/*A*t) versus time, Ao is the dye’s initial absorbance (at t = 0 min), and At is that absorbance at time = t.

#### Recycling and catalyst recovery

[Co(II)TP-OHPP]/CPTES−Bent/Cs **3** composite was recycled. After the completeness of the reaction, Simple filtering was employed to extract the supported catalyst from the reaction media, which were then washed with distilled water and utilized again for further studies. Then directly used in the next degradation reaction. Optimal circumstances were met in a series of catalytic degradation studies to examine the catalyst’s capacity for recycling. For these tests, it was the same sample used for these assays. The experiment was conducted with the following parameters set: 10 mL solution volume; initial (1.33 × 10^−4^ M) M.O dye, (1.11 × 10^−4^ M) M.R dye, and (1.42 × 10^−4^ M) OII dye concentration, 15 × 10^−3^g/ml of catalyst dosage, 8 × 10^−2^ M H_2_O_2_ concentration at 40 °C and the reaction time: 45, 135, 75 min for M.O dye, M.R dye, O II dye respectively. The degradation efficiency was investigated according to:

2 $$\text{Degradation}\:\text{efficiency}\:{\%}=\text{[(A}\circ-\text{A}_\text{t})/\text{A}_\text{0}]\times\text{100}$$

The dye’s absorbance at zero time is denoted by *A*_o_, and at time “t,” it is denoted by *A*_t_, at *λ*_max M.O_ = 464 nm, *λ*_max M.R_ = 505 nm, *λ*_max O II_ = 464 nm.

## Results and discussion

**Fig. 2:**
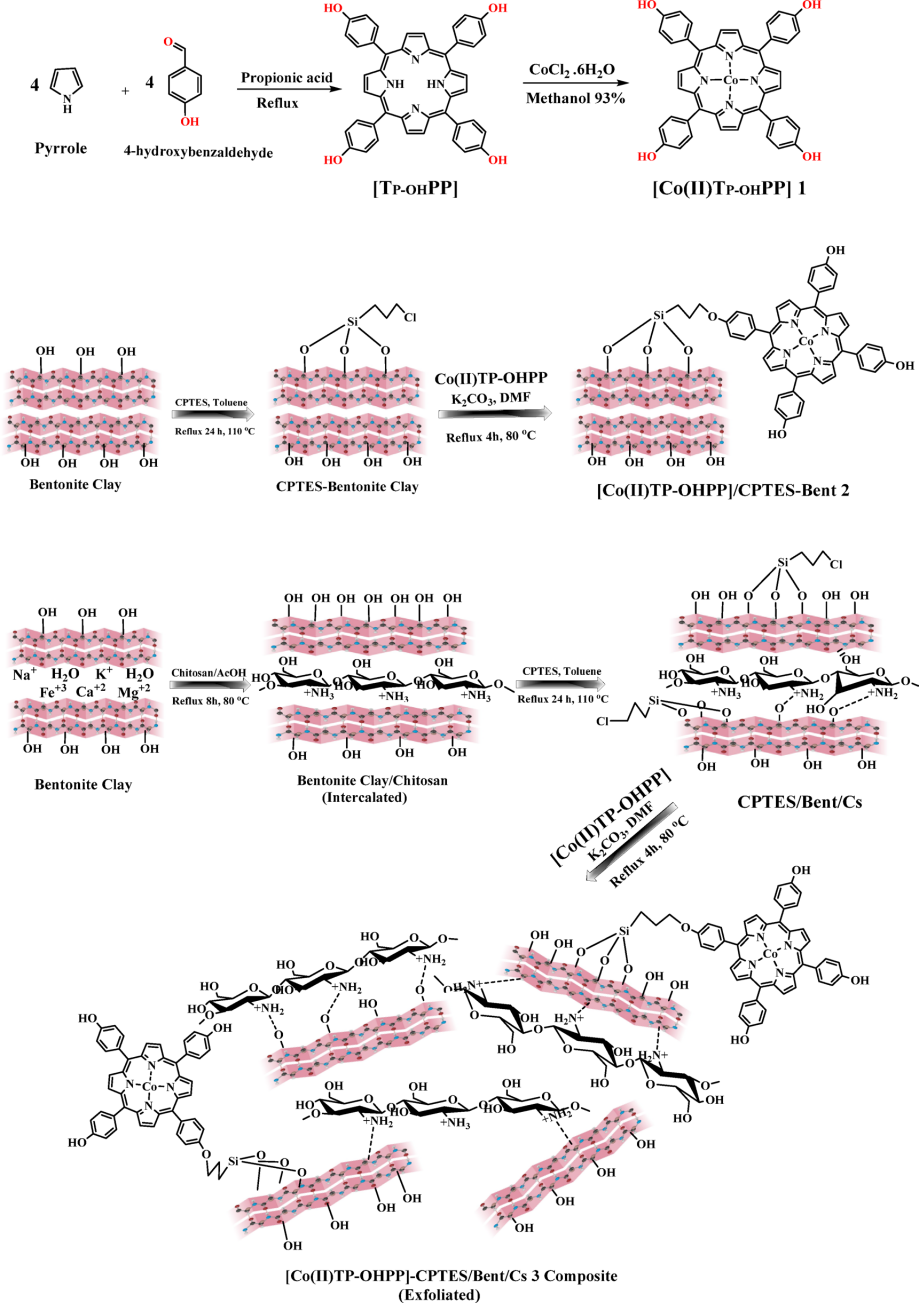
The schematic illustration of the prepared [Co(II)TP-OHPP] 1, [Co(II)TP-OHPP]/CPTES-Bent 2 and[Co(II)TP-OHPP]/CPTES−Bent/Cs 3 composite.

The preparation of 5,10,15,20 Tetrakis[4-(hydroxyl)phenyl]porphyrin [TP-OHPP] involved the condensation of 4-hydroxybenzaldehyde and pyrrole in propionic acid^43^, Metalation of porphyrin with CoCl_2_·6H_2_O in DMF forming [Co(II) TPHPP]**1**^44^. The chlorosilylation of bentonite clay with (3-chloropropyl)triethoxysilane in dry toluene occurs synchronously^45^. Ultimately, [Co(II)TP-OHPP] formed a covalent link with chlorosilylated bentonite clay creating [Co(II) TP-OHPP]/CPTES-Bent **2** through refluxing in the presence of potassium carbonate^46^. The bentonite clay undergoes a cationic modification using the chitosanium ion (Bent/Cs)^47^, followed by a surface modification using 3-chloropropyl triethoxy silane (CPTES)^45^. The ultimate chemical coupling of CPTES−Bent/Cs with the [Co(II) TP-OHPP] via refluxing when potassium carbonate is present forming [Co(II)TP-OHPP]/CPTES−Bent/Cs **3** composite^46^ as shown in Figure (2).

### Structural characterizations and morphologies analysis

#### FT-IR spectra

**Fig. 3:**
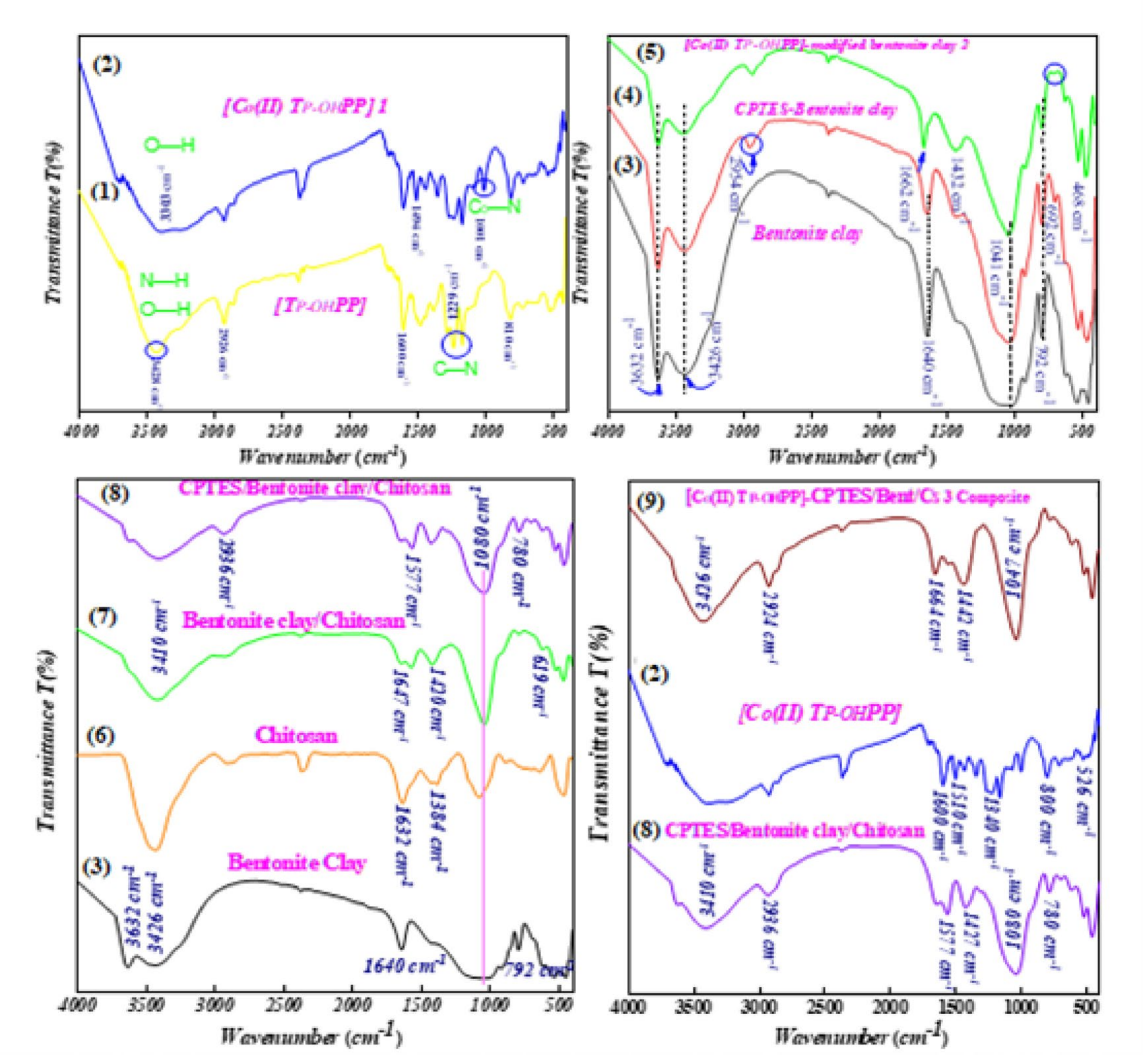
FT-IR spectra for (1) [TPHPP], (2) [Co(II) TPHPP] 1, (3) bentonite clay, (4) CPTES-bentonite clay, (5) [Co(II) TP-OHPP]/CPTES-bentonite clay 2, (6) chitosan, (7) bentonite clay/chitosan, (8) CPTES−bentonite clay/chitosan, (9) [Co(II)TP-OHPP]/CPTES−Bent/Cs 3 composite.

**Fig. 4:**
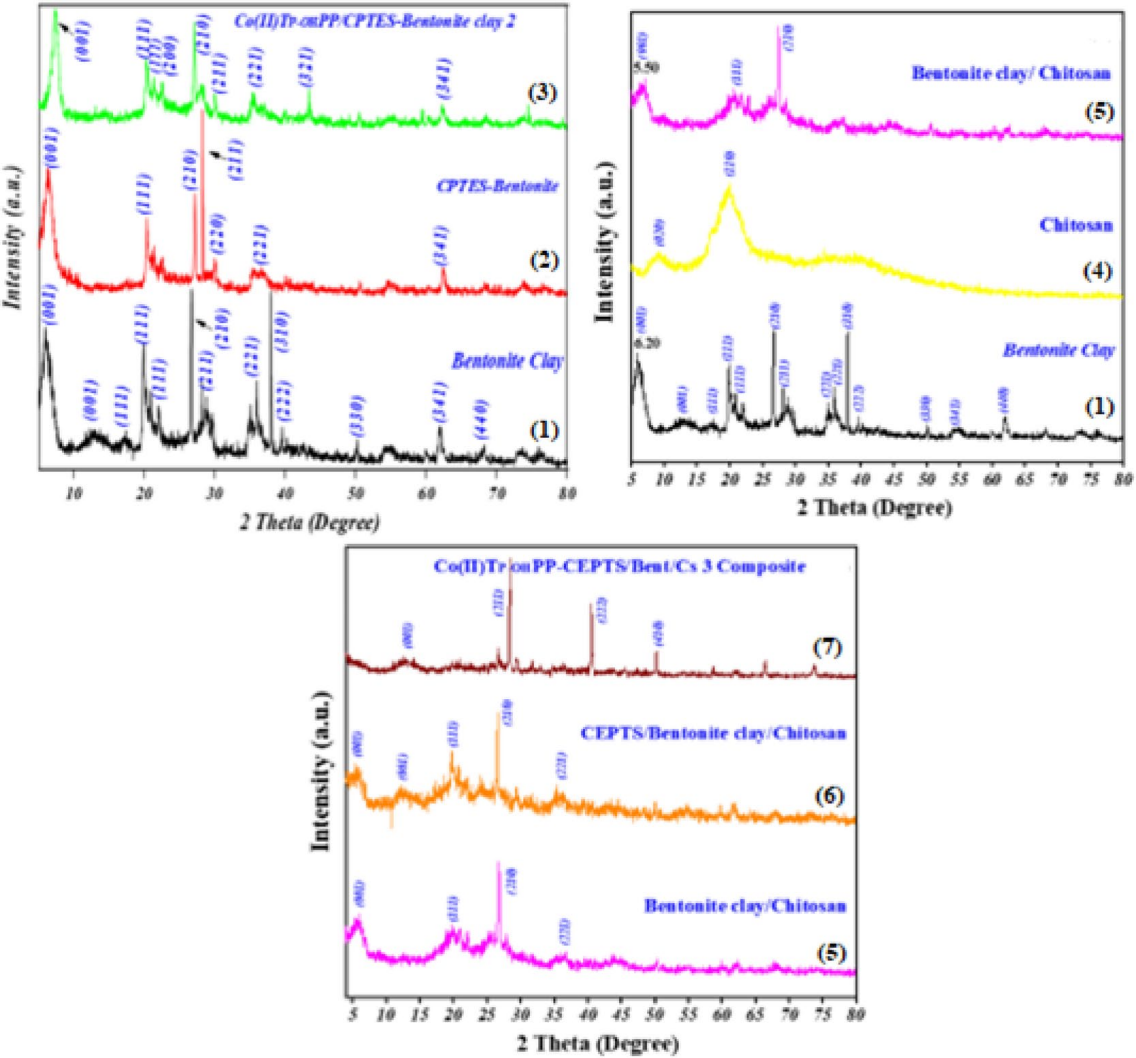
XRD patterns for (1) bentonite clay, (2) CPTES-bentonite clay, (3) [Co(II) TP-OHPP]/CPTES-bentonite clay 2 composite, (4) chitosan, (5) bentonite clay/chitosan, (6) CPTES−bent/Cs, (7) [Co(II)TP-OHPP]/CPTES−Bent/Cs 3 composite.

**Fig. 5:**
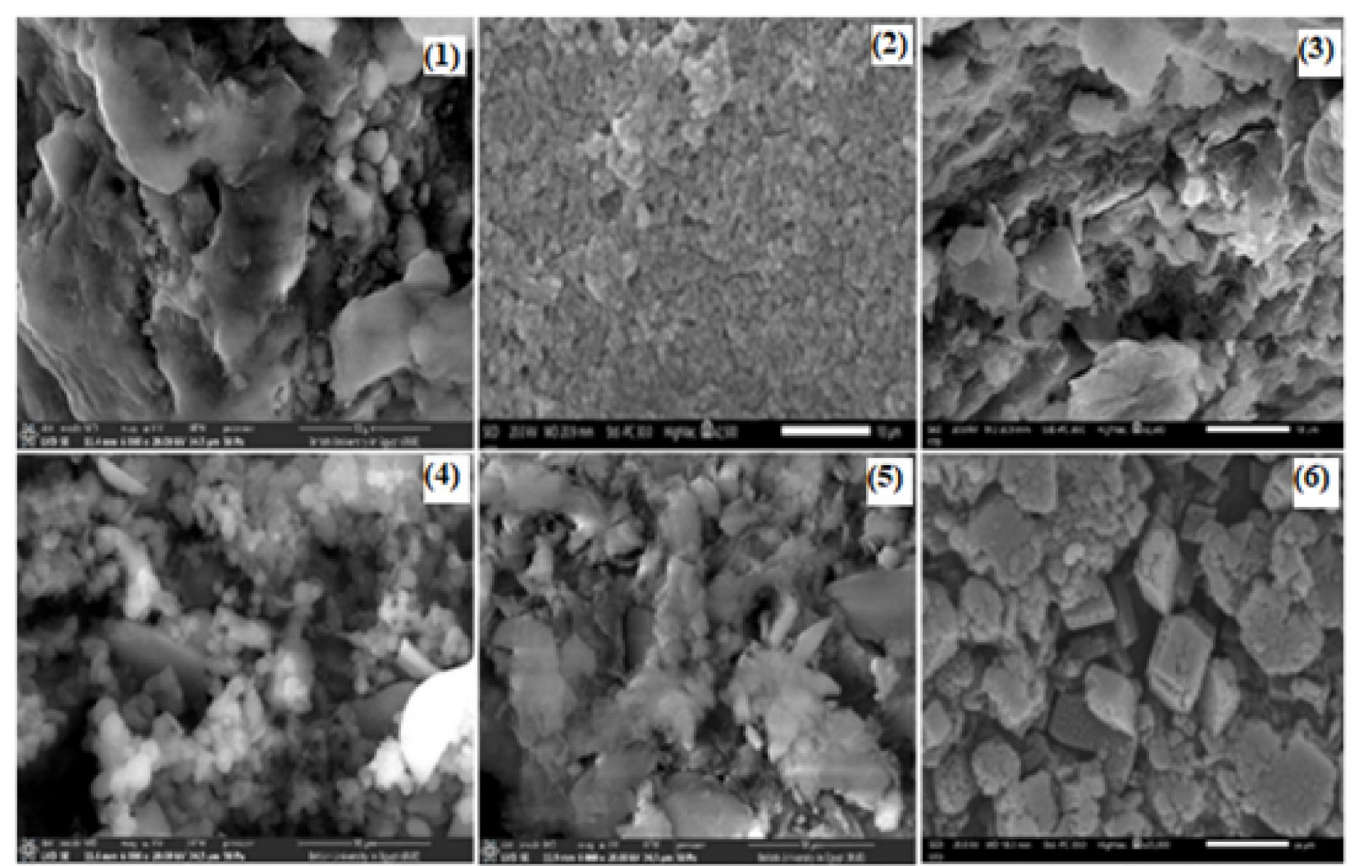
SEM image for (1) bentonite clay, (2) chitosan, (3) bentonite clay/chitosan, (4) [Co(II) TPHPP] 1, (5) [Co(II) TPHPP]/CPTES-bent 2, (6) [Co(II)TP-OHPP]/CPTES−Bent/Cs 3 composite.

**Fig. 6:**
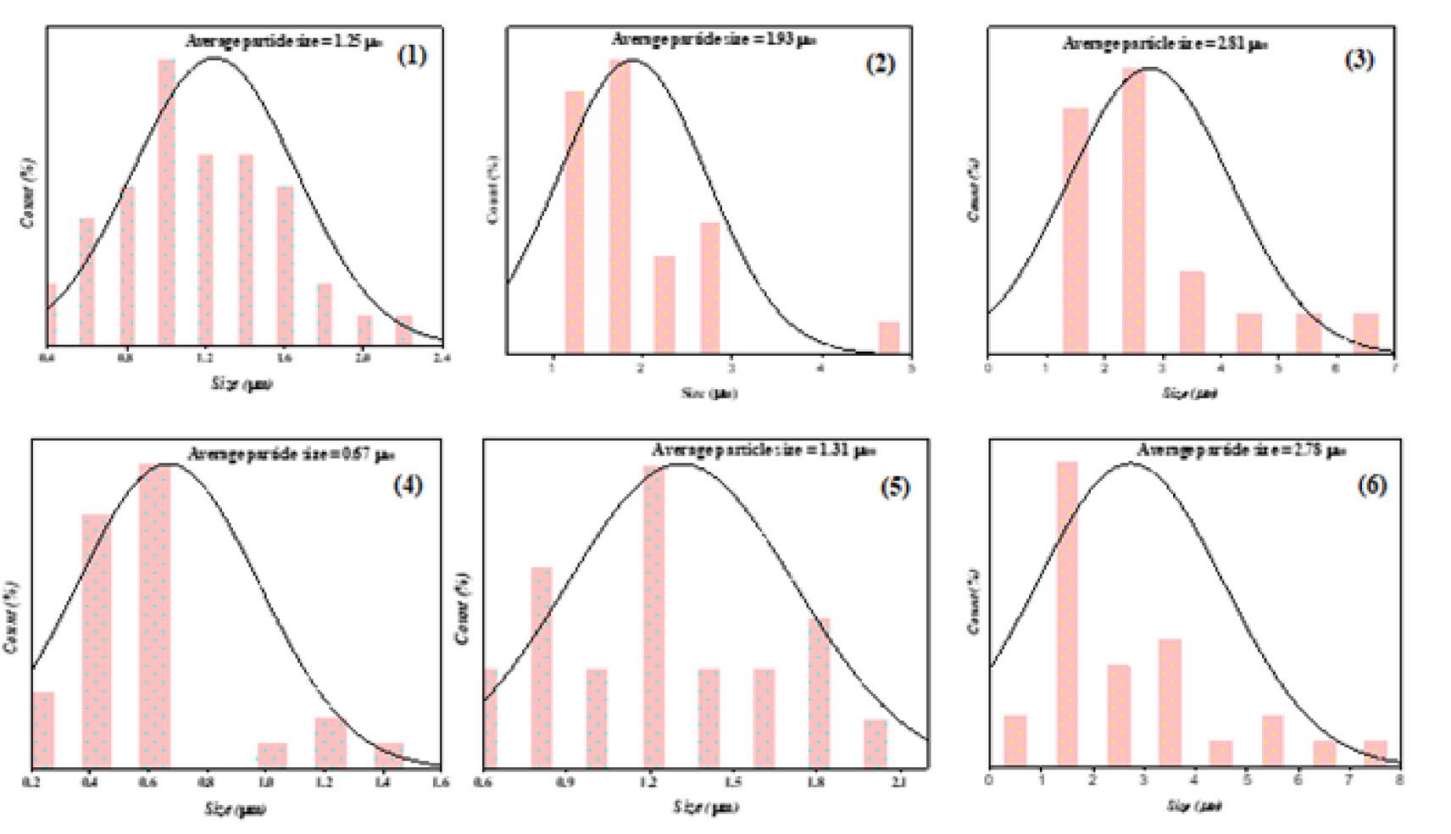
The histogram of the particle size distribution curve of the (1) Bentonite clay, (2) Chitosan, (3) Bentonite/Chitosan, (4) Co(II) Porphyrin complex, (5) [Co(II) TP-OHPP]-CPTES/Bentonite Composite, and (6) [Co(II) TP-OHPP]-CPTES-Bentonite clay/Chitosan Composite.

**Fig. 7:**
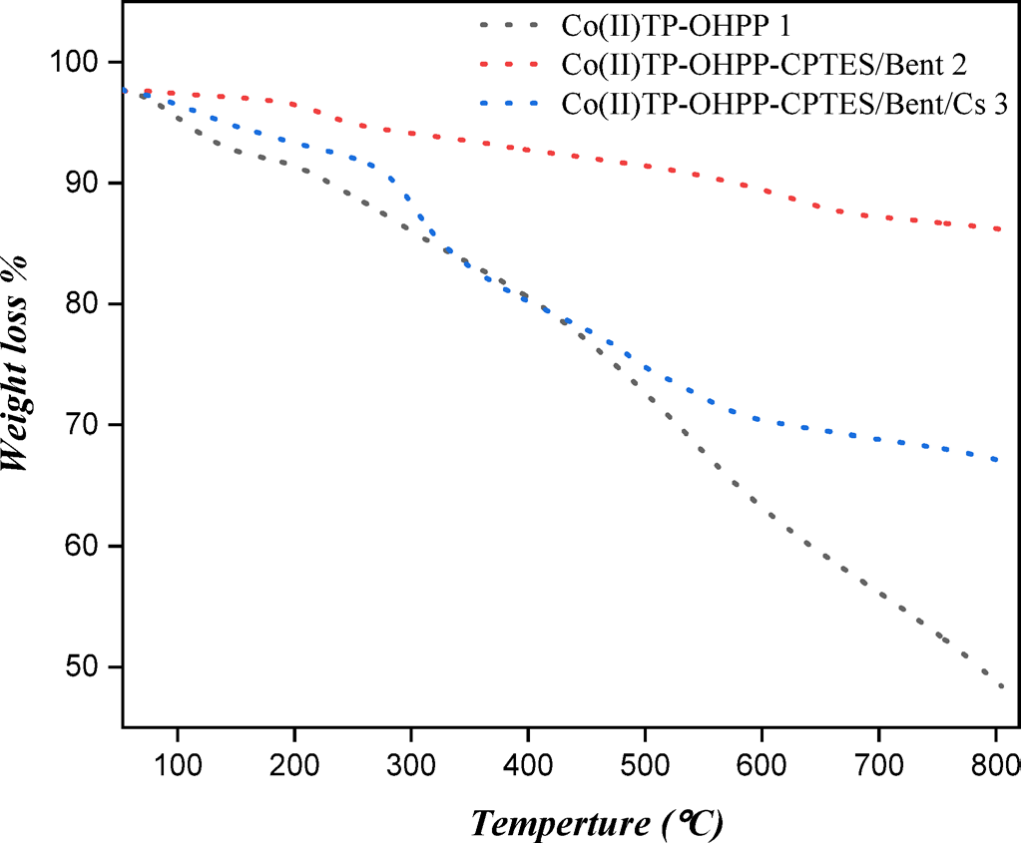
TGA analysis of [Co(II)TP-OHPP] 1, [Co(II)TP-OHPP]-CPTES/Bentonite clay composite, [Co(II)TP-OHPP]-CPTES/Bentonite clay/Chitosan 3 composite.

**Fig. 8:**
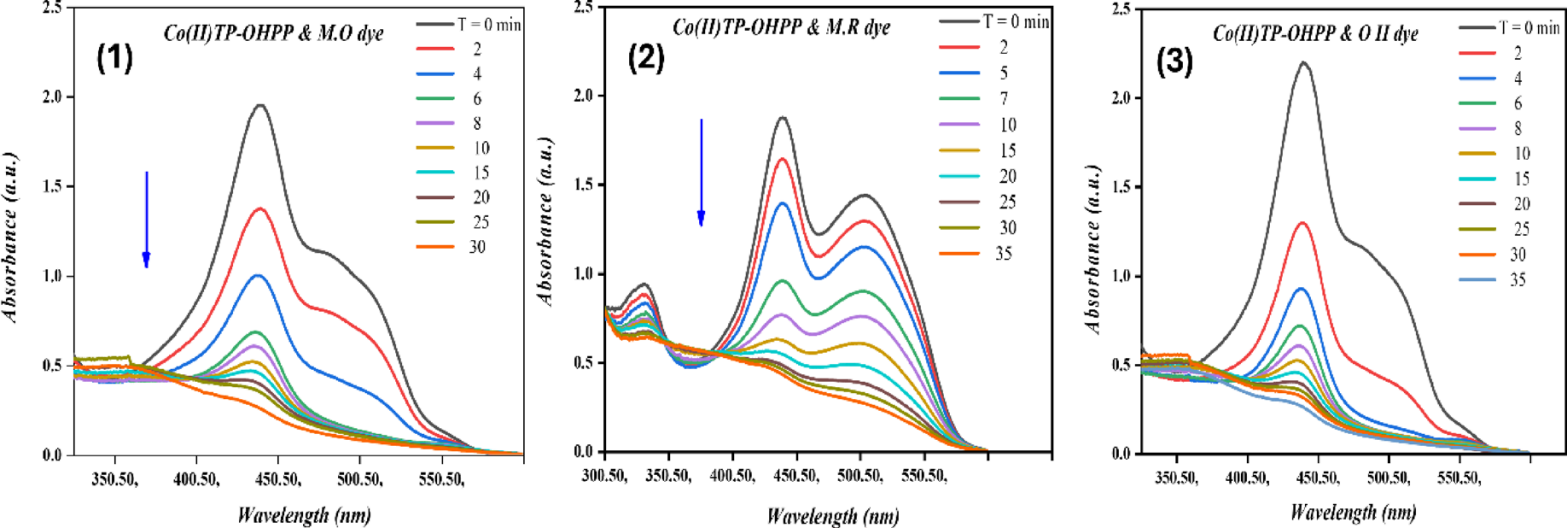
Electronic absorption spectra for decolorization of (8 − 1) M.O, (8 − 2) M.R, and (8 − 3) O II dyes using [Co(II) TP-OHPP] 1. For reaction conditions: (1.33 × 10^−4^ M) M.O dye, (1.11 × 10^−4^ M) M.R dye, (1.42 × 10^−4^ M) OII dye, H_2_O_2_ (8 × 10^−2^M) in the presence of (9.489 × 10^−4^mol/ml) of [Co(II)TP-OHPP] 1, pH = 9 at 40 °C.

**Fig. 9:**
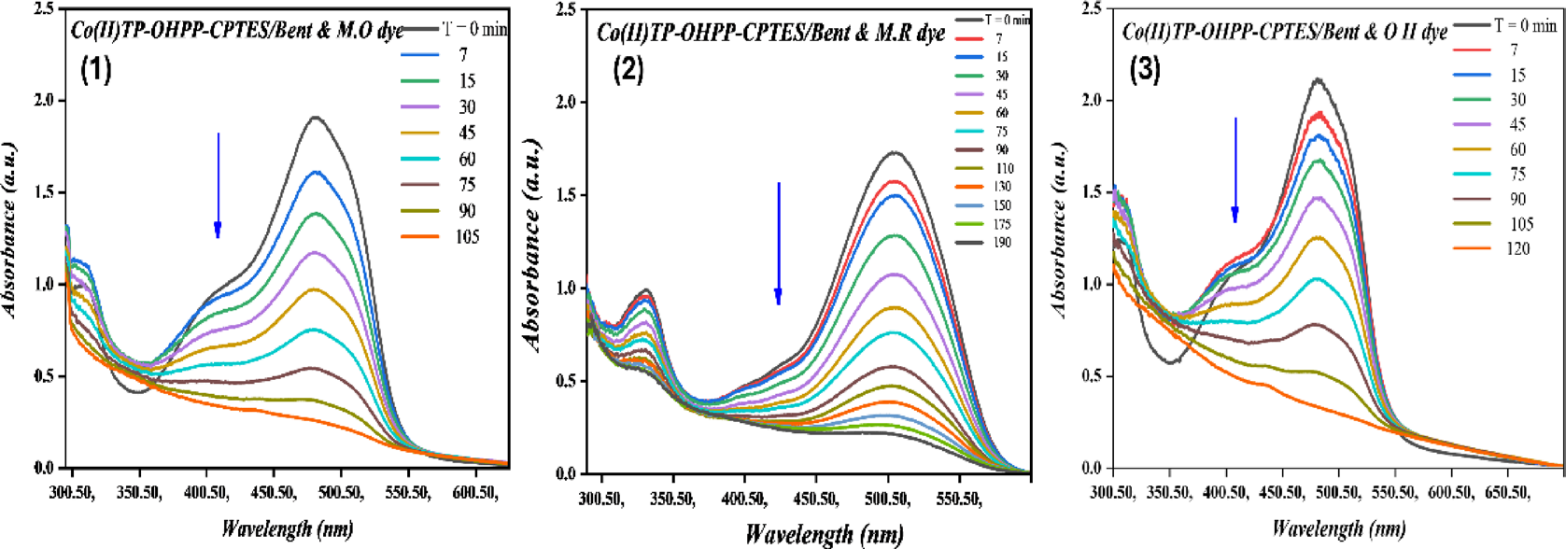
Electronic absorption spectra for decolorization of (9 − 1) M.O, (9 − 2) M.R, and (9 − 3) O II dyes using [Co(II)TP-OHPP]/CPTES-bent 2. For reaction conditions: (1.33 × 10^−4^ M) M.O dye, (1.11 × 10^−4^ M) M.R dye, (1.42 × 10^−4^ M) OII dye, H_2_O_2_ (8 × 10^−2^M) in the presence of (15 × 10^−3^g/ml) of [Co(II)TP-OHPP]/CPTES-bent 2, pH = 9 at 40 °C.

The FT-IR spectra of [TP-OHPP] is consistent with its chemical structure, as shown in Figure (3 − 1). The overlapped O−H stretching vibration with the N−H vibration is responsible for the band seen at around 3428 cm^−1^. Furthermore, stretching vibrations of the amine group C−N were identified by the peaks at 1228 and 1169 cm^−1^, and N–H bending and C = N vibrations were identified by the peaks at 1599 and 1469 cm^−1^, respectively. Ultimately, the N–H out-of-plane bending vibration of the macrocycle ring was identified as the source of the peak at 810 cm^−1^. [Co(II) TP-OHPP] **1** spectrum shows the N–H vibration frequency of free base porphyrin vanishes, and the phenol O–H stretching peak is indicated by a broad peak that is generated at 3303 cm^−1^ Figure (3 − 2)^43,48,49^. The newly created peak at 1001 cm^−1^ in Figure (3 − 2) represents the distinctive absorption of the Co–N(equatorial) bond established in [Co(II)TP-OHPP] **1**^27,50^. Bentonite clay illustrates in Figure (3–3), the stretching vibration of O−H of Bentonite and adsorbed water is responsible for the broad peak at ν 3632, 3426 cm^−1^. Approximately 1640 cm^−1^ of bending vibration indicates the existence of adsorbed water H–O–H. In the area ν 1000–1100 cm^−1^, Si–O–Si vibrations are responsible for the significant peak. At ν 792 cm^−1^ and ν 531 cm^−1^, respectively, their bending vibrations of Si–O and Al–O–Si produced peaks^45^. The spectra displayed peaks caused by bentonite, as seen in Figure (3–4). Additionally, the stretching vibration of the – CH_2_ – groups is responsible for a peak at 2954 cm^−1^. A fresh peak at 692 cm^−1^ verified that C–Cl was present in the CPTES group^51^. The IR band moves from 1641 cm^−1^ to 1662 cm^−1^ for the [Co(II) TP-OHPP]/CPTES-bentonite clay **2** spectrum Figure (3–5), suggesting a more constrained and ordered environment for adsorbed water molecules. In contrast to environments with fluctuating hydrogen bonds, this shift indicates decreased water mobility and more stable hydrogen bonding, producing unique vibrational modes that imply better order and stability around the water molecules^52,53^, covalent anchoring of the [Co(II) TP-OHPP] molecules to the bentonite−Cl is demonstrated by the peak at 692 cm^−1^ disappearing and another new band formed at 1432 cm^−1^. The spectrum of [Co(II) TP-OHPP]/CPTES-Bentonite **2** showed no distinctive bands for [Co(II) TP-OHPP] since the spectra of [Co(II) TP-OHPP] and CPTES-bentonite overlapped throughout the infrared spectrum^54^. Chitosan’s spectra Figure (3–6) display a large band at v 3426 cm^−1^, which is where the intermolecular H−bond induced stretching vibration of the O−H and N−H groups is located. The C = O stretching vibration (amide band) and the C−N stretching vibration in amide are represented by the absorption bands at ν 1632 cm^−1^ and 1384 cm^−1^. Additionally, the C−O stretching vibration is represented by the band at ν 1080 cm^−1^^55^. Figures (3–7) shows Strong hydrogen bonding is indicated by the broad O–H and N–H bands shifting from ν 3632, 3426 cm^−1^ to 3410 cm⁻^1^ when positively charged chitosan (^+^NH₃) and negatively charged bentonite contact. At 2924 cm^−1^, an aliphatic C–H stretch extending from chitosan is seen. The partly acetylated chitosan’s C = O stretching, N–H bending and C–N stretching are represented by the bands at 1647, 1577, and 1420 cm^−1^. At 1080 cm^−1^, a strong peak for Si–O–Si vibration is seen, whereas at 619 cm^−1^, Si–O–Al bending is noted^47^. Bentonite clay/chitosan caused peaks in the spectra, as shown in Figure (3–8). The stretching vibration of the – CH_2_ – groups also caused an intense peak at v 2936 cm^−1^. In the CPTES group, the existence of C–Cl was verified by a new peak at ν 780 cm^−1^. These bands provide confirmation of the successful grafting of CPTES onto the bentonite clay/chitosan^45^. As demonstrated by the [Co(II)TP-OHPP]/CPTES−Bent/Cs **3** composite spectrum Figure (3–9), hydrogen bonding between the metalloporphyrin and the clay/chitosan causes shifts in the O-H and N-H stretching to ν 3426 cm⁻^1^. The band at ν 1600 cm⁻^1^ was displaced to ν 1664 cm^−1^, and aliphatic C–H stretches from chitosan emerged at ν 2924 cm^−1^ applied from propyl chain and chitosan. The interactions of metalloporphyrin with the clay/chitosan composite caused the C = N, C = C stretches at ν 1577 cm⁻^1^ to shift to ν 1442 cm⁻^1^. At ν 1074 cm⁻^1^, Si–O stretching from bentonite clay and C–O stretching from chitosan developed, while the peak at ν 780 cm⁻^1^ vanished, indicating that the Co(II)TP-OHPP molecules were covalently bonded to the CPTES−bentonite/chitosan^46^.

#### XRD analysis

The XRD pattern of bentonite clay is displayed in Figure (4 − 1), where the peaks represent the layers of silicate and metal cations arranged in an organized manner. Tetrahedral sheets of silica sandwiched between octahedral sheets containing metal cations make up the layered structure. The (001) lattice plane’s characteristic 2θ value was 6.20°, with a d-spacing of 1.42 nm. The existence of water molecules and exchangeable cations between the layers, as well as the layers’ spacing, all have a significant impact. The well-ordered crystallographic planes of bentonite clay are reflected by other sharper peaks at higher angles that correspond to different interplanar spacings^56^. The pattern for CPTES-modified bentonite clay is depicted in Figure (4 − 2), where some of the significant peaks found in the pristine bentonite clay are still present but are less intense. Some of the peaks for bentonite clay disappeared at 2θ = 13.01°, 17,28°, 37,92°. This might be because the silane molecules grafted onto the clay surface, possibly obscuring or partially disrupting the crystalline structure. The general pattern suggests that while the crystallinity has not been completely destroyed by the alteration process, the structure has been somewhat changed^57^. The pattern for [Co(II)TP-OHPP]/CPTES-modified bentonite clay **2** is displayed in Figure (4 − 3), which also demonstrates further alterations in comparison to the earlier samples. The overall pattern is more diffuse, and the peaks’ sharpness has been greatly diminished, suggesting a further decrease in crystallinity. Because the cobalt porphyrin complex contains large organic molecules that could disrupt the normal crystalline lattice of bentonite clay, it probably adds more disorder to the structure. A mix of crystalline and amorphous phases is suggested by the existence of smaller peaks or broad humps, suggesting that the complex may be dispersed throughout the modified clay matrix^58^. An extensive peak at 2θ = 9.60° can be seen in the XRD patterns of chitosan chains Figure (4–4). Indicates amorphous crystals and a more intense peak at 2θ = 20.22°, which shows the somewhat regular arrangement of chitosan chains as a result of diffraction from the (020) and (110) planes of the crystalline lattice, which have interplane lengths of 0.92 nm and 0.438 nm, respectively. Interestingly, the crystalline index was 55, and the corresponding degree of acetylation was 81%^59–62^. As seen in Figure (4–5), the primary (001) peak of bentonite clay 2θ = 6.20° migrated towards a lower, broadening diffraction angle at 2θ = 5.50°, indicating an increase in interlayer spacing to d = 15.7 Å. This drop is explained by a deformation of the regularity silicate layers as well as a crystallinity distortion brought on by the chitosan chain insertion. At 9.27°, 12.27°, 28.84°, 35.01°, and 37.94°, some peaks for bentonite and chitosan vanished, while others interfered at 19.8°. picture demonstrates that chitosan strands have intercalated into the bentonite clay layers^63,64^. The mean (001) peak in Figure (4–6) is further broadened and its intensity is decreased, indicating that CPTES has been modified with surface hydroxyl groups and intercalated inside the bentonite clay layers. chitosan and CPTES, two big organic compound modifications, increase structural chaos. At 2θ = 12°, a new (001) peak emerged, signifying the formation of siloxane linkages between CPTES molecules and the clay surface^57^. The disappearance of lattice planes (001), (111) at 5.5°, and 19.90° in Figure (4–7) indicates that the addition of metalloporphyrin complex has caused the bentonite layers to become more disordered and completely exfoliated. Other peaks emerged at 40.6°, and 50.2°, which are attributed to the formation of new organized structure morphology^58^. Lattice parameters of Co(II)TP-OHPP/CPTES–bentonite clay **2** composite and Co(II)TP-OHPP/CPTES-Bent/Cs **3** composite are shown in Table S1.

#### Scanning electron microscope (SEM)

Scanning electron microscopy (SEM) images of four distinct materials are shown in Fig. 5: (1) Bentonite clay, (2) chitosan, (3) bentonite clay/chitosan, (4) [Co(II)TP-OHPP] **1**, and (5) [Co(II)TP-OHPP]/CPTES-bentonite clay **2**, (6) [Co(II)TP-OHPP]/CPTES−Bent/Cs **3** composite. Each image demonstrates the materials morphological traits and particle size distribution, offering insight into how surface alterations and complicated integration affect the structure at the microscale.

The structure of bentonite clay in Figure (5 − 1) displays an extremely dense and compact matrix, characterized by irregular, asymmetrical particle aggregates. The characteristic low permeability of these aggregates is a result of the microscopic particles that appear to be packed closely together. The apparent texture shows a layered structure, typical of phyllosilicate minerals, with some smooth patches and uneven regions indicating areas of variable porosity, which is typical of clay minerals like bentonite. There is a heterogeneous particle size distribution in the image, as evidenced by the bigger clusters and smaller particles scattered throughout. The SEM image of chitosan displays an irregularly dispersed granule pattern on a rough, irregular surface Figure (5 − 2). Additionally, it has aggregated structures, where particles group together to enhance the uneven surface morphology, and micropores, which give it a porous seems. The bentonite clay/chitosan shows in Figure (5 − 3), where the spacing between bentonite layers increases, opening up the dense, compact matrix of the original bentonite. The result is an expanded structure with greater porosity, and the chitosan molecules also coat the surface of bentonite particles, forming a smoother, more uniform outer layer. In comparison to untreated bentonite, this surface coating creates a more continuous morphology by filling in some of the natural roughness and imperfections. In contrast to the bentonite clay, bentonite clay/chitosan samples. The [Co(II)TP-OHPP] **1** shows in Figure (5 − 4) exhibits a radically different morphology. Granular-looking particles that are much smaller and more homogeneous are visible in the image. A clear particulate structure characteristic of organic or organometallic compounds is indicated by the particles dense packing and decreased overall cohesiveness when compared to the clay samples. The granular texture and uniformity suggest that the cobalt porphyrin complex produces uniformly small particles due to its molecular structure. Features from the cobalt porphyrin complex and the modified bentonite clay are combined in the [Co(II)TP-OHPP]/CPTES-bentonite clay **2** composite Figure (5–5). Compared to the [Co(II)TP-OHPP] complex alone, the structure seems more cohesive, producing larger aggregates while preserving some of the granular texture visible in the [Co(II)TP-OHPP] image. The resultant composite material shows a heterogeneous morphology that reflects both the organic complex and the changed clay, this indicates that the cobalt complex has been successfully incorporated into the clay matrix. [Co(II)TP-OHPP]/CPTES−Bent/Cs **3** composite material illustrated in Figure (5–6), exhibits a group of particles with a variety of morphologies, including irregular shapes, flat surfaces, and sharp edges. The particles flat, planar surfaces and sharp edges raise the prospect that they have a crystalline structure, which is a property of materials with a well-organized atomic structure. Surface interactions between the modified bentonite clay/Cs and the cobalt porphyrin complex may be the cause of these behaviours. Figure 6 shows the histogram of the particle size distribution curve of (1) bentonite clay, (2) chitosan, (3) bentonite clay/chitosan, (4) [Co(II) TPHPP] **1**, (5) [Co(II) TPHPP]/CPTES-bentonite clay **2** composite, (6) [Co(II)TP-OHPP]/CPTES−Bent/Cs **3** composite.

#### Thermogravimetric analysis (TGA)

Figure 7 shows the weight loss (%) as a function of temperature (°C) ranging from about 50 °C to 800 °C for three samples including: Co(II) TP-OHPP **1**, Co(II) TP-OHPP -CPTES/bentonite clay composite **2**, and Co(II) TP-OHPP -CPTES/bentonite clay/chitosan composite **3.** The Co(II) porphyrin complex **1** exhibits a weight loss between (~ 100–800 °C), indicating decomposition of the organic framework. The low residual mass (~ 40–45%) at 800 °C suggests minimal inorganic content, consistent with the presence of only the metal center as a stable oxide and the absence of any supporting material. While the metalloporphyrin-CPTES/bentonite clay **2** composite shows the slowest degradation process, with weight loss initiating at a temperature (~ 200 °C). The higher residual weight at 800 °C (~ 85–90%) reflects the presence of thermally stable bentonite clay, which acts as an inorganic support. The clay matrix improves thermal stability by restricting porphyrin chain mobility and providing a partial thermal barrier, thereby delaying decomposition and enhancing overall thermal resistance. The TGA graph for Co(II) porphyrin-CPTES/bentonite clay/chitosan **3** composite exhibits a multi-step weight loss. The first stage (~ 100–350 °C) corresponds to the decomposition of chitosan, while the second stage (~ 350–600 °C) is attributed to the thermal degradation of the metalloporphyrin complex, leaving a high residual mass (~ 65–70%) at 800 °C due to the thermally stable inorganic clay matrix. However, in comparison to the Co(II) porphyrin porphyrin-CPTES/bentonite clay **2** composite, the overall thermal stability is lower. The compact, thermally stable network that forms between CPTES, bentonite, and metalloporphyrin is disrupted by the addition of chitosan, a thermally less stable biopolymer, which adds more breakdown sites. The interaction of several organic and inorganic components also results in structural heterogeneity, which reduces interfacial bonding and causes heat deterioration to start earlier. By limiting molecular mobility and functioning as a heat barrier, this inorganic support delays the breakdown of the organic components^65–68^.

#### Zeta potential and dynamic light scattering (DLS) analysis

##### Zeta potential analysis

Table 1 presents the zeta potential data for bentonite clay, which exhibits strongly negative values ranging from − 29.3 to − 24.9 mV. This high negative surface charge is characteristic of natural clays, due to their negatively charged silicate layers. Such a surface charge indicates strong electrostatic repulsion forces that help prevent molecular aggregation. Functionalization of bentonite with the Co(II) porphyrin complex and CPTES significantly reduces the negative surface charge, with zeta potential values ranging from − 13.3 to − 13.1 mV. This shift indicates successful surface modification, likely resulting from partial neutralization and surface coverage by the organ silane (CPTES) and the porphyrin complex. These modifications likely shield the negatively charged sites on the bentonite surface, thereby decreasing electrostatic repulsion among particles. While the [Co(II)TP-OHPP]/CPTES–Bent/Cs **3** composite shows a further reduces the zeta potential to approximately − 6.4 to − 6.8 mV. This significant decrease reflects a shift toward a more electropositive (or less negatively charged) surface, primarily due to the presence of protonated amino groups in chitosan, a natural polycationic biopolymer. These positively charged groups interact with the negatively charged sites on the bentonite surface, leading to partial neutralization of surface charge.

**Table 1:** Zeta potential of bentonite clay, [Co(II)TP-OHPP]/CPTES- bentonite clay composite, and [Co(II)TP-OHPP]/CPTES- bent/Cs composite.

No	Sample	Zeta potential (mV)		
1	Bentonite Clay	−29.3	−26.9	−24.9
3	[Co(II)TP-OHPP]/CPTES- Bentonite Clay	−13.3	−13.2	−13.1
4	[Co(II)TP-OHPP]/CPTES- Bent/Cs	−6.4	−6.8	−6.4

##### Average particle size

Table 2 shows the average particle size of [Co(II) TP-OHPP] **1**, [Co(II) TP-OHPP]/CPTES-bentonite clay **2** and [Co(II)TP-OHPP]/CPTES−Bent/Cs **3** according to DLS measurements. The particle size gradually increased from 0.63 nm for the Co(II) porphyrin complex to 1.64 nm following its incorporation into CPTES-modified bentonite clay. Due to interfacial interactions, the interaction between the metalloporphyrin and the silane-modified clay surface causes a higher hydrodynamic size, which is the reason for this rise. When the bentonite was further modified by cationic functionalization with chitosan, the particle size rose to 2.55 nm. Because of the high molecular weight natural polymer chitosan, which covers and integrated within the particles and promotes further aggregation and network creation, the apparent particle size has significantly increased.

**Table 2:** Average particle size of [Co(II)TP-OHPP] 1, [Co(II)TP-OHPP]-CPTES/Bentonite clay 2, [Co(II)TP-OHPP]-CPTES/Bentonite clay/chitosan 3 composite.

No	Sample	Average size, d (µm)
1	[Co(II)TP-OHPP] 1	0.63
2	[Co(II)TP-OHPP]/CPTES-Bentonite Clay 2	1.64
3	[Co(II)TP-OHPP]/CPTES-Bentonite clay/Chitosan 3	2.55

### Catalytic oxidation of M.O, M.R, and O II dyes using [Co(II) TP-OHPP] 1, [Co(II)TP-OHPP]/CPTES-bentonite clay 2, and [Co(II)TP-OHPP]/CPTES −bent/cs 3 composite

The catalytic oxidation of (M.O, M.R, and O II) has been investigated using hydrogen peroxide as an oxidant and [Co(II)TP-OHPP] **1**, [Co(II) TP-OHPP]/CPTES−Bent **2** composite, [Co(II)TP-OHPP]/CPTES−Bent/Cs **3** composite as catalysts in aqueous solution. The oxidation reaction was followed by recording the UV–vis spectra of the reaction mixture with time, the collapse of the main absorbance bands of the reported three dyes at λ_max_ = 464 nm for M.O dye, λ_max_ = 505 nm for M.R dye, and λ_max_ = 485 nm for O II almost vanished. Figure 8-(1, 2, and 3), illustrates the superior catalytic performance of the soluble [Co(II)TP-OHPP] **1** complex, achieving 95% degradation of the three studied dyes within 30 min for M.O, and 35 min for both M.R and O II.

The supported [Co(II) TP-OHPP]/CPTES−Bent **2**, shows a breakdown of 95% of the three dyes that were reported in 105 min for M.O, 190 min for M.R, and 120 min for O II dye, as shown in Fig. 9-(1, 2, and 3).

**Fig. 10:**
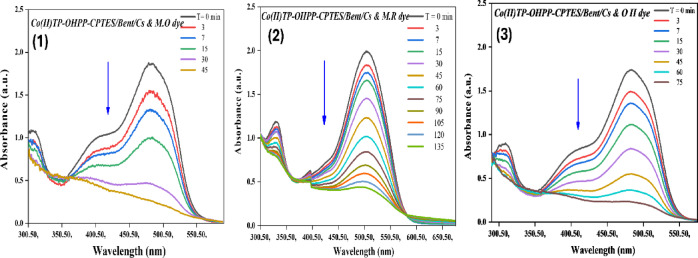
Electronic absorption spectra for decolorization of (10 − 1) M.O, (10 − 2) M.R, and (10 − 3) O II dyes using [Co(II)TP-OHPP]/CPTES−Bent/Cs 3 composite. For reaction conditions: (1.33 × 10^−4^ M) M.O dye, (1.11 × 10^−4^ M) M.R dye, (1.42 × 10^−4^ M) OII dye, H_2_O_2_ (8 × 10^−2^M) in the presence of (15 × 10^−3^g/ml) of [Co(II)TP-OHPP]/CPTES−Bent/Cs 3 composite, pH = 9 at 40 °C.

Figure 10-(1, 2, and 3) represents the degradation percent of the reported three dyes reached 95% within 45 min for M.O, 135 min for M.R, and 75 min for O II using [Co(II)TP-OHPP]/CPTES−Bent/Cs **3** composite as catalyst. The conjugated system in the structures of all three anionic dyes was destroyed, resulting in colorless oxidation products.

The kinetic rate for the destruction of the three anionic dyes displayed in (Table 3). The data indicates that the degradation of M.O, M.R, and O II was regarded as a first-order rate kinetic^69,70^, which can be expressed simply as ln A_o_/A_t_ = k_obs_ t. and the First-order plot for the degradation of MO, MR and OII dyes are shown in Figure S1,S2, and S3.

**Table 3:** Kinetic reaction rate for M.O, M.R, and O II dyes degradation with various catalysts.

Catalyst	Dye type	Rate constant (min^−1^)	Degradation time (min)
[Co(II) TP-OHPP] 1	M.O	0.167	30
	M.R	0.103	35
	O II	0.135	35
[Co(II)TP-OHPP]/CPTES-Bent 2	M.O	0.011	105
	M.R	0.006	190
	O II	0.008	120
[Co(II)TP-OHPP] −CPTES/Bent/Cs 3 composite	M.O	0.051	45
	M.R	0.023	135
	O II	0.011	75

The homogenous cobalt porphyrin [Co(II)TP-OHPP] **1** shows the highest efficiency in catalytic degradation of the three dyes, this efficiency is attributed to the unique structure of cobalt porphyrin in facilitating the catalytic reaction by maximizing the contact between the catalyst, H_2_O_2,_ and the dye molecules. Despite [Co(II)TP-OHPP] **1** complex having an outstanding catalytic activity suffering from its susceptibility to self-decomposition, losing its active sites, being toxic owing to its solubility, and can’t separate from the reaction mixture. Subsequently, overcoming the limitations associated with this homogenous catalyst, we immobilized the soluble [Co(II)TP-OHPP] **1** on to bentonite clay to form [Co(II) TP-OHPP]/CPTES−Bent **2** composite and also supported onto bentonite clay/chitosan as shown in [Co(II)TP-OHPP]/CPTES−Bent/Cs **3** composite, resulted in more stable, reusable catalyst with no toxicity in the degradation process of the three reported anionic dyes.

As shown in Table 3, [Co(II)TP-OHPP]/CPTES−Bent/Cs **3** composite, shows the highest supported catalyst in the degradation process of the three anionic dyes due to the cationic alteration of bentonite clay by chitosanium ions is responsible for the increased rate of oxidative degradation. This change greatly increases catalytic effectiveness by facilitating closer interaction between hydrogen peroxide, anionic dye molecules, and the supported catalyst’s active sites. So, we investigated some further analyses on M.O, M.R, and O II dyes in the presence of the [Co(II)TP-OHPP]/CPTES−Bent/Cs **3** composite because of its benefit and uniqueness.

### Mineralization and proposed degradation pathway of M.O, M.R, O II dyes

Analysis of total organic carbon (TOC) is a crucial technique for assessing the mineralization of dye oxidative degradation events. Under typical reaction conditions at 40 °C, oxidation of M.O, M.R, O II dyes using [Co(II)TP-OHPP]−CPTES/Bent/Cs **3** composite revealed elimination of 69%, 85%, and 66% after 45, 135, and 75 min, respectively, suggesting inadequate mineralization of the dye to CO_2_ and H_2_O as shown in (Table 4).

**Table 4:** TOC analysis for M.O, O II, and M.R dyes before and after oxidative degradation process.

Dye type	TOC before cracking (PPM)	TOC after cracking (PPM)	Degradation efficiency percentage (%)	Notes
Methyl orange	60.8	18.8	69%	Substantial decrease in TOC levels following degrading experiments
Methyl red	61.6	9.12	85%	
Orange II	42	14.28	66%	

As indicated in (Table 5), gas chromatography-mass spectrometry (GC–MS) analysis was used to examine the degradation behaviour of M.O, M.R, and O II and identify the residual organic intermediate products at various retention times in order to further assess the effectiveness of the catalytic Co(II)TP-OHPP]−CPTES/Bent/Cs **3** composite with an H_2_O_2_ system.

**Table 5:**
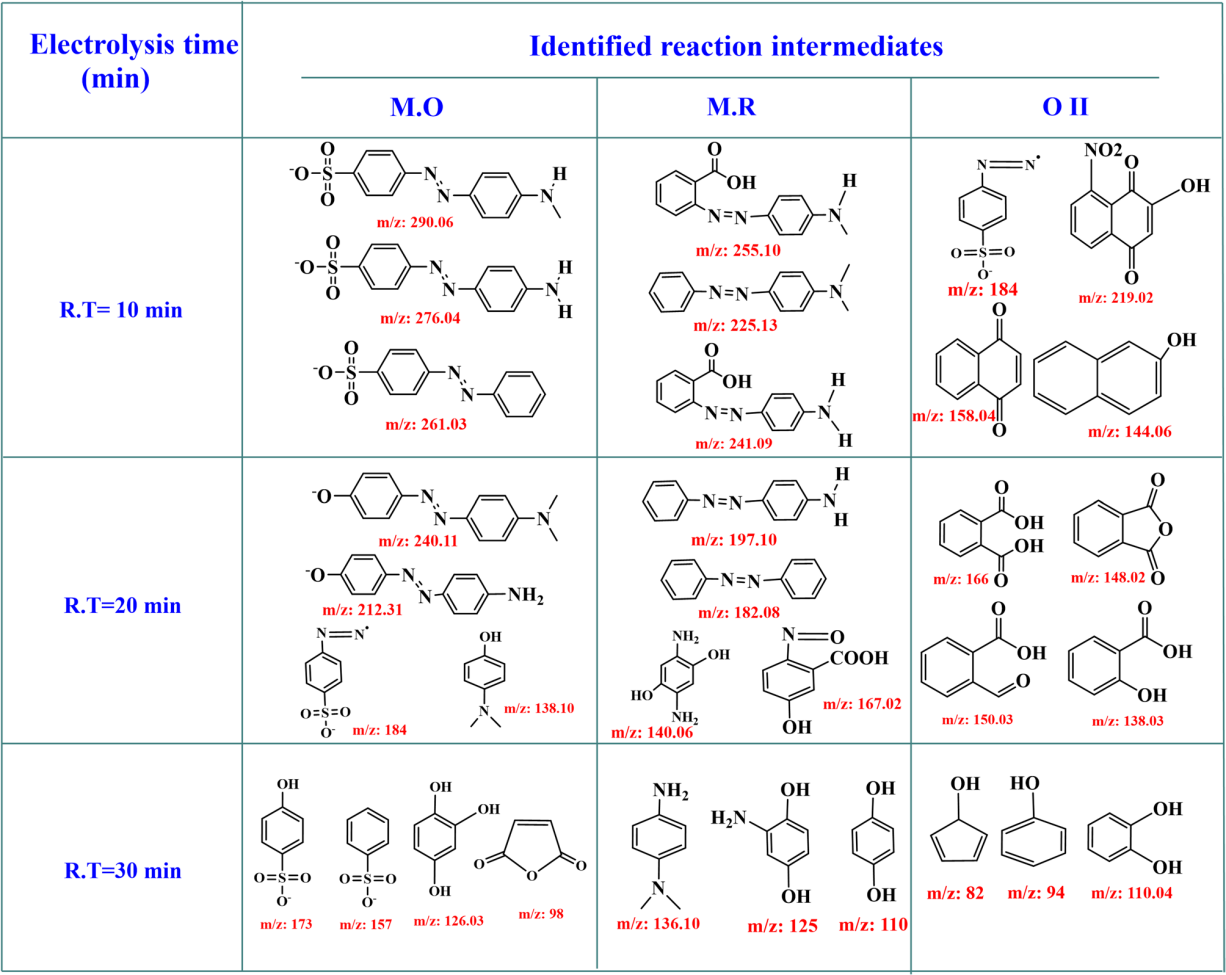
GC-Mass analysis for M.O dye, M.R dye, and O II dye after oxidative degradation process.

The degradation of M.O, M.R, and O II dyes produces several common intermediate products, as reported in the literature. M.O dye degradation produces compounds such as (E)−4-((4 (methylamino)phenyl) diazenyl) benzenesulfonate, (E)−4-((4-amino phenyl) diazenyl) benzenesulfonate, (E)−4-(phenyldiazenyl)benzenesulfonate, (E)−4-((4-(l1-oxidaneyl) phenyl) diazinyl)-N, N-dimethylaniline, (E)−4-((4-(l1-oxidaneyl) phenyl) diazenyl) aniline, 4-hydroxybenzenesulfonic acid, furan-2,5-dione, and various hydroxylated and sulfonated derivatives^71–73^.

Similarly, M.R dye degradation results in intermediates like (E)−2-((4-(methylamino) phenyl)diazenyl)benzoic acid, (E)−2-((4-aminophenyl) diazenyl) benzoic acid, (E)−4-(phenyldiazenyl)aniline, 5-hydroxy-2-nitrosobenzoic acid, 2,5-diaminobenzene-1,3-diol, N,N-dimethylbenzene-1,4-diamine, quinol, and other aromatic amines^74–76^.

The breakdown of O II dye results in products such as 4-(diazenyl) benzenesulfonate, 2-hydroxy-8-nitronaphthalene-1,4-dione, naphthalene-1,4-dione, phthalic acid, 2-formylbenzoic acid, 2-hydroxybenzoic acid, isobenzo furan-1,3-dione, catechol, cyclopenta-2,4-dien-1-ol^21,77^. These findings highlight the common pathways and intermediates in the degradation of azo dyes.

### Recovery and recycling of catalyst

**Fig. 11:**
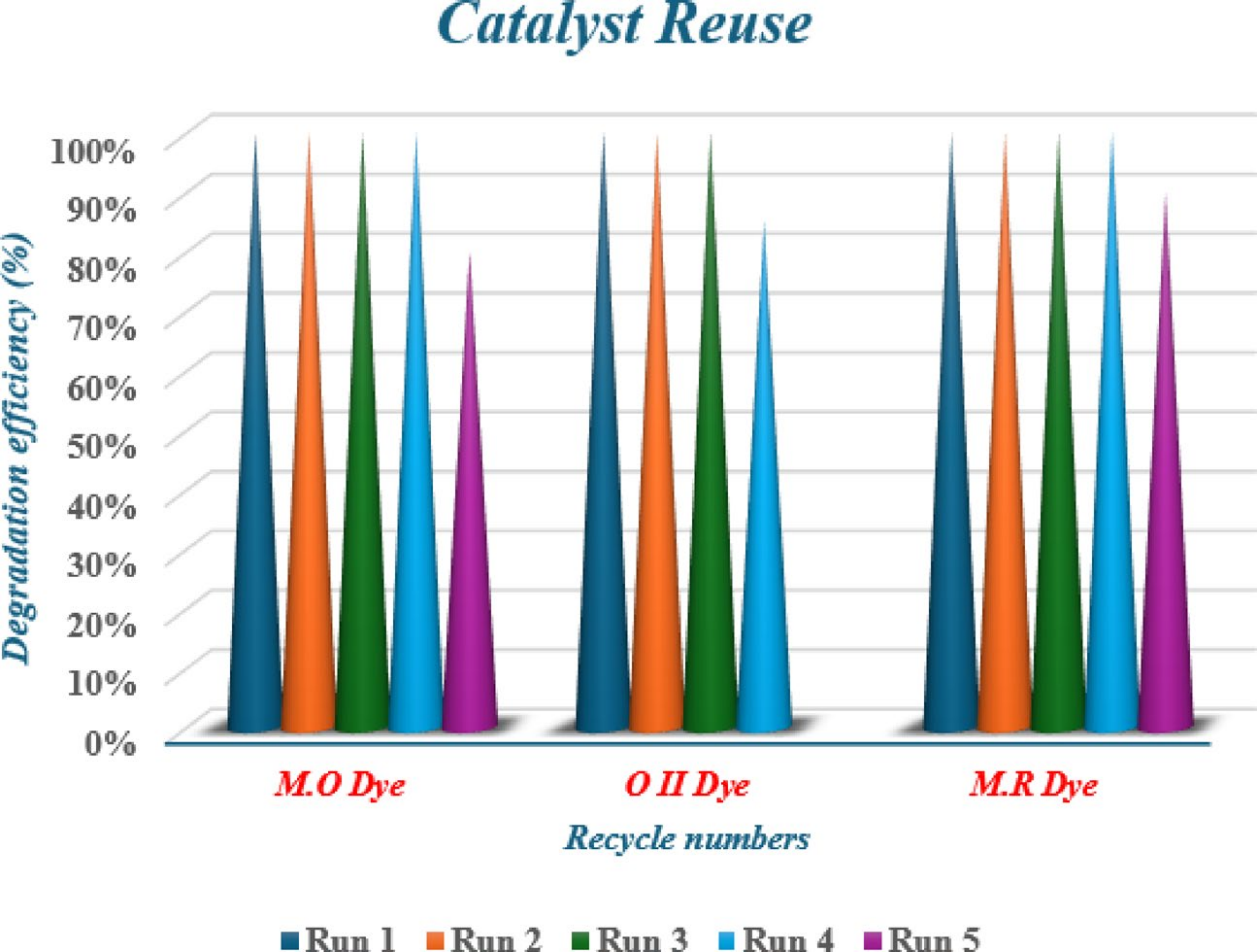
Effect of [Co(II)TP-OHPP]−CPTES/Bent/Cs 3 composite recycling on the degradation of M.O, O II, and M.R dyes. Catalyst 15 × 10^−3^g/ml; [M.O]: 1.32 × 10^−4^ M, [O II]: 1.42 × 10^−4^ M, [M.R]: 1.11 × 10^−4^ M, [H_2_O_2_]: 8 × 10^−2^ M, pH:9 at 40 °C.

There are numerous benefits to using heterogeneous catalysts in the catalytic oxidation process, such as reduced reaction costs, decreased waste generation, and the creation of more cost-effective and environmentally responsible separation and recycling methods. The [Co(II)TP-OHPP]−CPTES/Bent/Cs **3** composite was readily removed from the solution by straightforward filtration and repurposed for further reactions after being repeatedly rinsed with distilled water. Figure 11 shows the percentages of degradation for the three dyes M.O, O II, and M.R.

The degradation efficiency of M.O, M.R, and O II dyes was evaluated following several cycles (Table 6). For the first four cycles of M.O degradation, the [Co(II)TP-OHPP]/CPTES−Bent/Cs **3** composite performed steadily; however, in the fifth cycle, its activity dropped by 20%. Similarly, the catalyst activity for O II degradation remains constant for three cycles before declining by 15% in the fourth. Four cycles of stability were noted in the case of M.R deterioration, with the fifth cycle showing an 8% decrease in activity.

**Table 6:** Effect of [Co(II)TP-OHPP]/CPTES−Bent/Cs 3 composite recycling on the degradation efficiency of M.O, O II, and M.R dyes.

Recycle numbers	M.O Dye D.E(%)	O II Dye D.E (%)	M.*R* Dye D.E (%)
Run 1	100%	100%	100%
Run 2	100%	100%	100%
Run 3	100%	100%	100%
Run 4	100%	85%	100%
Run 5	80%		92%

**Fig. 12:**
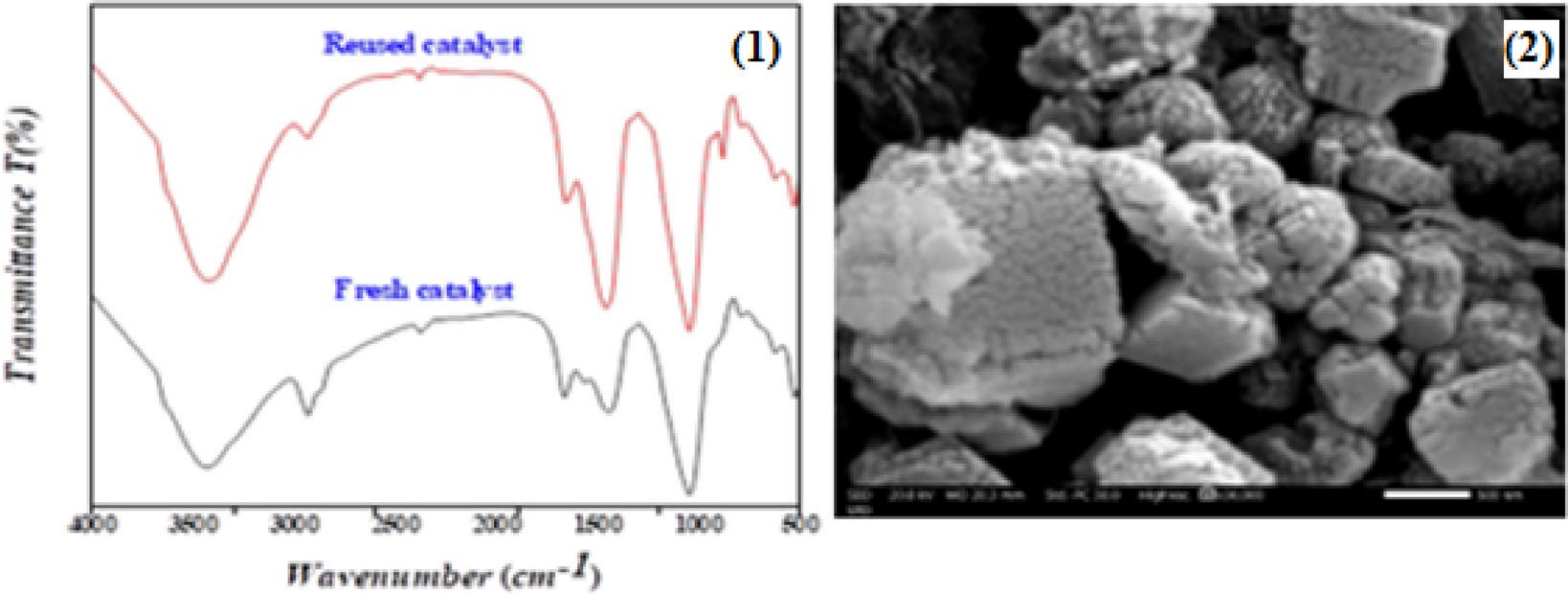
(1) FTIR spectrum, and (2) SEM for [Co(II)TP-OHPP]/CPTES−Bent/Cs 3 composite after five cycles for dye removal from the solution.

The FT-IR of the [Co(II)TP-OHPP]/CPTES−Bent/Cs **3** composite after five cycles is shown in the spectrum at Figure (12 − 1). During the reusability experiments, the chemical structure of the Co(II)TP-OHPP]/CPTES−Bent/Cs **3** composite does not change since the functional groups of both the Co(II) porphyrin and CPTESBent/Cs stay intact. Figure (12-2) a illustrates the surface morphology after five cycles of reusability of [Co(II)TP-OHPP]/CPTES−Bent/Cs **3**. The image reveals a well-preserved structure, indicating that the composite maintains their integrity and do not exhibit significant degradation or morphological changes despite repeated use.

Table 7 provides a comparative analysis of our designed system [Co(II)TP-OHPP]/CPTES−Bent/Cs **3** composite and other previously published results against various dyes degradation under different conditions found in the literature. [Co(II)TP-OHPP]/CPTES−Bent/Cs **3** composite gave higher degradation yield and mineralization with a shorter reaction time required and also showed high catalytic stability compared to the other heterogeneous catalysts used in the comparison.

**Table 7:** Comparison of [Co(II)TP-OHPP]/CPTES−Bent/Cs 3 composite with other heterogenous **systems.**

Catalyst	Abbreviations	Conditions	Efficiency	References
Nickel(II) meso-tetra(aryl) porphyrin used in catalytic degradation of methyl orange (M.O) dyes with H_2_O_2_.	[Ni(TAMPP)]	M.O degradation was carried out at [Ni(TAMPP)] m = 5 mg (0.00488 mmol), the H_2_O_2_ aqueous solution Co = 2 ml.L^−1^, pH = 6 and dye concentrations of 30 mg/L, T = 293 K.	The catalyst shows degradation 77% of M.O dye within 90 min.	^78^
Mn(III) porphyrins supported onto multi-walled carbon nanotubes in the green oxidation of methyl orange dye with H_2_O_2_.	MnTCPPOAc@ MWCNT	The experimental conditions: 3.3 mL of 500 mg/L aqueous M.O solution, solid catalyst (1.99.10^–4^ mmol) at 25 °C, H_2_O_2_ (5 mmol).	The mineralization rate was about 46% within 24 h.	^30^
Polysaccharide/Fe(III)-porphyrin hybrid film as catalyst for oxidative decolorization of methyl orange (M.O), methyl red (M.R) dyes.	Cht/PVA-FeTMPyP	The experimental conditions of each reaction were set as: catalyst dosage = 60 mg; solution volume = 25 mL; initial dye concentration= 10 ppm; pH = 7; H_2_O_2_ concentration = 1 mmol/L; room temperature.	The catalyst shows 100% of M.O and 92% of M.R degraded within 90 min.	^28^
Oxidative degradation of methyl orange dye by using magnetically recyclable nanocatalyst based on manganese-porphyrin.	[Fe_3_O_4_@SiO_2_@NH_2_@(Mn(TCPP)OAc)]	The experimental conditions: 0.014 mmol of M.O solution, solid catalyst (6.28 × 10^−4^ mmol), pH 3 at 45 °C, H_2_O_2_ (14.99 mmol).	The magnetically nanocatalyst revealed 76% of M.O dye degraded within 5 h.	^79^
Catalytic degradation of acid orange 7 dye using cobalt (II) porphyrin complex supported on chitosan/graphene oxide nanocomposite.	[Co(II) TPHPP]- Cs/GO	The experiments were carried out at (1.42 × 10^−4^M) AO7 dye, with H_2_O_2_ (8 × 10^−2^M) in the presence of [Co(II) TPHPP]- Cs/GO nano composite (15 × 10^−3^g/ml) and pH = 9 at 40 °C.	Removal efficiency of AO7 94%, within 60 min. TOC shows 50%dye mineralization.	^43^
chitosan/MMT biocomposite in the Sono assisted adsorption removal of orange II dye.	[chitosan/MMT biocomposite]	The experiments were carried out under condition of at 1 g/L [chitosan/MMT], 200 mg/L [AO7], 350 W ultrasonic power, and pH 6.95.	Removal efficient 51.8% within time: 60 min.	^39^
Cobalt (II) complex of 5,10,15,20 tetrakis-[4-(hydroxy)phenyl] porphyrin complex incorporated onto CPTES−bentonite clay, CPTES− bentonite clay/chitosan composites for green oxidation of different anionic dyes.	[Co(II)TP-OHPP]/CPTES−Bent/Cs	The reaction conditions: (1.33 × 10^−4^ M) M.O dye, (1.11 × 10^−4^ M) M.R dye, (1.42 × 10^−4^ M) OII dye, H_2_O_2_ (8 × 10^−2^M) in the presence of (15 × 10^−3^g/ml) of catalyst, pH = 9 at 40 °C.	[Co(II)TP-OHPP]/CPTES−Bent/Cs achieved 95% removal of M.O, M.R, and O II in 45, 135, and 75 min, respectively. TOC revealed 69%, 85%, and 66% mineralization. High stability was observed with no significant change up to 5 cycles (M.O, M.R) and 4 cycles (O II).	The present work

### Identification of reactive oxygen species (ROS) through hydroxyl radical analysis using isopropyl alcohol

**Fig. 13:**
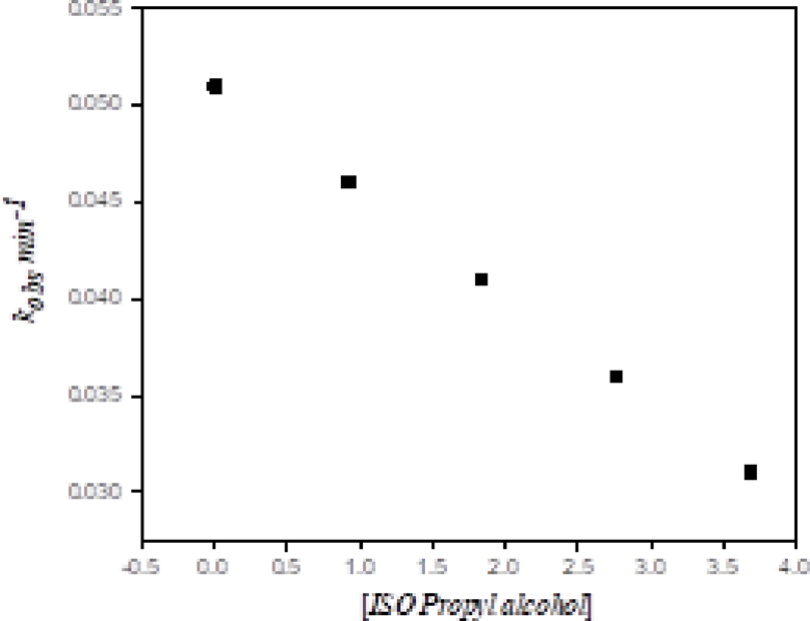
Effect of increased isopropyl alcohol on the decolorization of MO dye.

We have studied the inhibiting effect of isopropyl alcohol as a hydroxyl radical scavenging agent on the oxidation reaction of M.O dye by H_2_O_2_/catalyst because hydroxyl radicals were able to be the reactive species in the oxidation of the M.O dye by our catalytic system [Co(II)TP-OHPP]/CPTES−Bent/Cs composite/H_2_O_2_. As shown in (Fig. 13), the rate of degradation of dye was inhibited by increasing the concentration of isopropyl alcohol in the reaction solution. This indicates that decolorization of M.O by [Co(II)TP-OHPP]/CPTES−Bent/Cs composite/H_2_O_2_ involved the formation and participation of ^•^OH radicals as the active species in the oxidation reaction.

### Suggested mechanism

According to the following equation scheme, the oxidation reaction is thought to be caused by activating the H_2_O_2_ molecule, which results in the creation of hydroxyl radicals (^•^OH).

Where the metal ions are Co(II), the ligand is [TP-OHPP], and the supported CPTES−Bent/Cs is (S). The most effective catalyst for cobalt is a heterogeneous system. The breakdown of various hues and H_2_O_2_ has been facilitated by the use of cobalt as an activator due to its Fenton-type reactions. The hydroxyl radicals, •OH, are formed when the catalyst activates the H_2_O_2_ molecules, according to the proposed mechanism. By attacking the three dyes, the latter creates an active intermediate that breaks down in the rate-determining phase to produce the final oxidation product.

3 $$\text{S-[Co(II)TP-OHPP]+H}_2\text{O}_2\rightarrow^\bullet\text{OH}+^-\text{OH+S-[Co(III)TP-OHPP]}$$

4 $$\text{S-[Co(III)TP-OHPP]+}^-\text{OH}\rightarrow\text{S-[Co(II)TP-OHPP(OH)]}\rightarrow\text{S-[Co(II)TP-OHPP]}+^\bullet\text{OH}$$

5 $$\text{Dye}+^\bullet\text{OH}\rightarrow\text{Residuel}\:\text{organic}\:\text{intermediate}+\text{H}_2\text{O}_2+\text{CO}_2$$

## Conclusions

[Co(II)TP-OHPP] **1**, [Co(II)TP-OHPP]/CPTES−Bentonite clay composite **2**, [Co(II)TP-OHPP]/CPTES−Bentonite clay/Chitosan **3** composite were created, described, and utilized as a homogenous and solid catalyst to accelerate the green oxidative breakdown of M.O, M.R, and O II dyes in aqueous solution using hydrogen peroxide. The homogenous [Co(II)TP-OHPP] **1**, exhibits a high rate but has some limitations, including its failure to separate from the reaction mixture and the inability to be reused. The supported [Co(II)TP-OHPP]-CPTES/Bent **2** performance and capacity for oxidative deterioration were minimal, and the reusability tests were subpar, displaying its inadequate long-term efficiency and stability. The recoverable and resilient [Co(II)TP-OHPP]/CPTES−Bent/Cs **3** composite demonstrated high catalytic activity and decomposed 95% of the three dyes that were reported in 45 min for M.O, 135 min for M.R dye, 75 min for O II. Following the remarkable outcomes of using the solid [Co(II)TP-OHPP]/CPTES−Bent/Cs **3** composite, a study was carried out using total organic carbon analysis (TOC), which revealed that M.O, M.R, and O II dyes were oxidized, revealing the elimination of 69%, 85%, and 66% mineralization after 45, 135, and 75 min, respectively, under mild reaction conditions of (1.33 × 10^−4^ M) M.O dye, (1.11 × 10^−4^ M) M.R dye, (1.42 × 10^−4^ M) O II dye with H_2_O_2_ (8 × 10^−2^M) in the presence of [Co(II)TP-OHPP]/CPTES−Bent/Cs **3** composite (15 × 10^−3^g/ml) and pH = 9 at 40 °C. All of the degradation products of M.O, M.R, and O II dyes with varying retention durations were investigated by GC-MS studies. It is noteworthy that the performance of the recovered catalyst did not significantly degrade even after five cycles with M.O and M.R dyes and four cycles with O II dye of reuse. This innovation demonstrates the catalyst’s ability to effectively and sustainably address the problems associated with water contamination.

## Supplementary Information

Below is the link to the electronic supplementary material.

Supplementary Material 1

## Data Availability

Data are however available from the corresponding authors upon reasonable request.
