# Supplementary material for: Efficient oxidative degradation of Azo dyes by cobalt(II) porphyrin complex supported on modified bentonite and chitosan: structural characterization and mechanistic insight
Source: Sci Rep. 2025 Oct 1;15:34282. doi: 10.1038/s41598-025-19788-9 (PMC12488868; doi:10.1038/s41598-025-19788-9)
Supplement: Supplementary file 1 — Supplementary Material 1 [file 41598_2025_19788_MOESM1_ESM.docx]

**Efficient Oxidative Degradation of Azo Dyes by Cobalt(II) Porphyrin Complex Supported on Modified Bentonite and Chitosan: Structural Characterization and Mechanistic Insight**

**Sahar H. El‑Khalafy *, Mahmoud T. Hassanein, Nehal A. Salahuddin, & Mohamed M. Alaskary**

Department of Chemistry, Faculty of Science, University of Tanta, Tanta 31527, Egypt.

** Corresponding authors: Sahar H. El-Khalafy E-mail: sahar­_hasouna@science.tanta.edu.eg, Tel: +20-1007276665*

**Supplementary Materials**

| Lattice Parameters | Co(II)TP-OHPP/ CPTES-Bentonite clay | Co(II)TP-OHPP/ CPTES-Bent/Cs |
| --- | --- | --- |
| Lattice constant  *a* (Å) | **7.84** | **7.67** |
| Lattice volume  *V* (Å^3^) | **481.89** | **451.22** |
| Crystallite size  *D* (nm) | **38.74** | **16.40** |
| Dislocation line density *(⸹)* | **33.78** | **66.6** |
| Root means square error (RMS) | **19.50** | **22.84** |

**Table S1. Lattice parameters of Co(II)TP-OHPP/CPTES–bentonite clay 2 composite and Co(II)TP-OHPP/ CPTES-Bent/Cs 3 composite.**


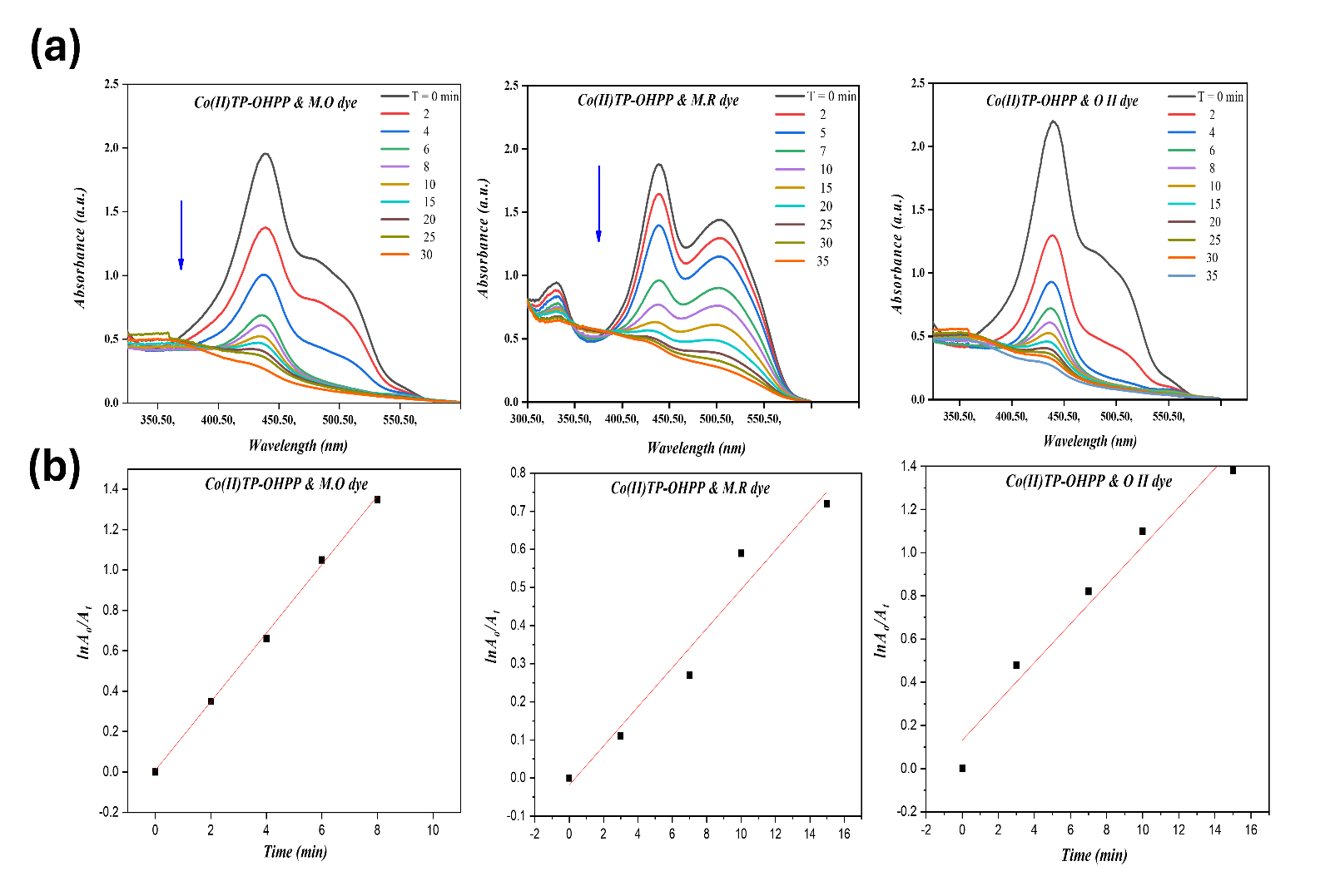

**Figure S1. (a) Electronic absorption spectra during decolorization of MO, MR and OII dyes using Co(II)TP-OHPP 1 (b) First-order plot for the degradation of MO, MR and OII with (k_obs_ = 0.17, 0.05 and 0.08 respectively). For reaction conditions: (1.33×10^−4^ M) M.O dye, (1.11×10^−4^ M) M.R dye, (1.42×10^−4^ M) OII dye, H_2_O_2_ (8×10^-2^M) in the presence of (9.489×10^-4^mol/ml) of [Co(II)TP-OHPP] 1, pH =9 at 40 °C.**


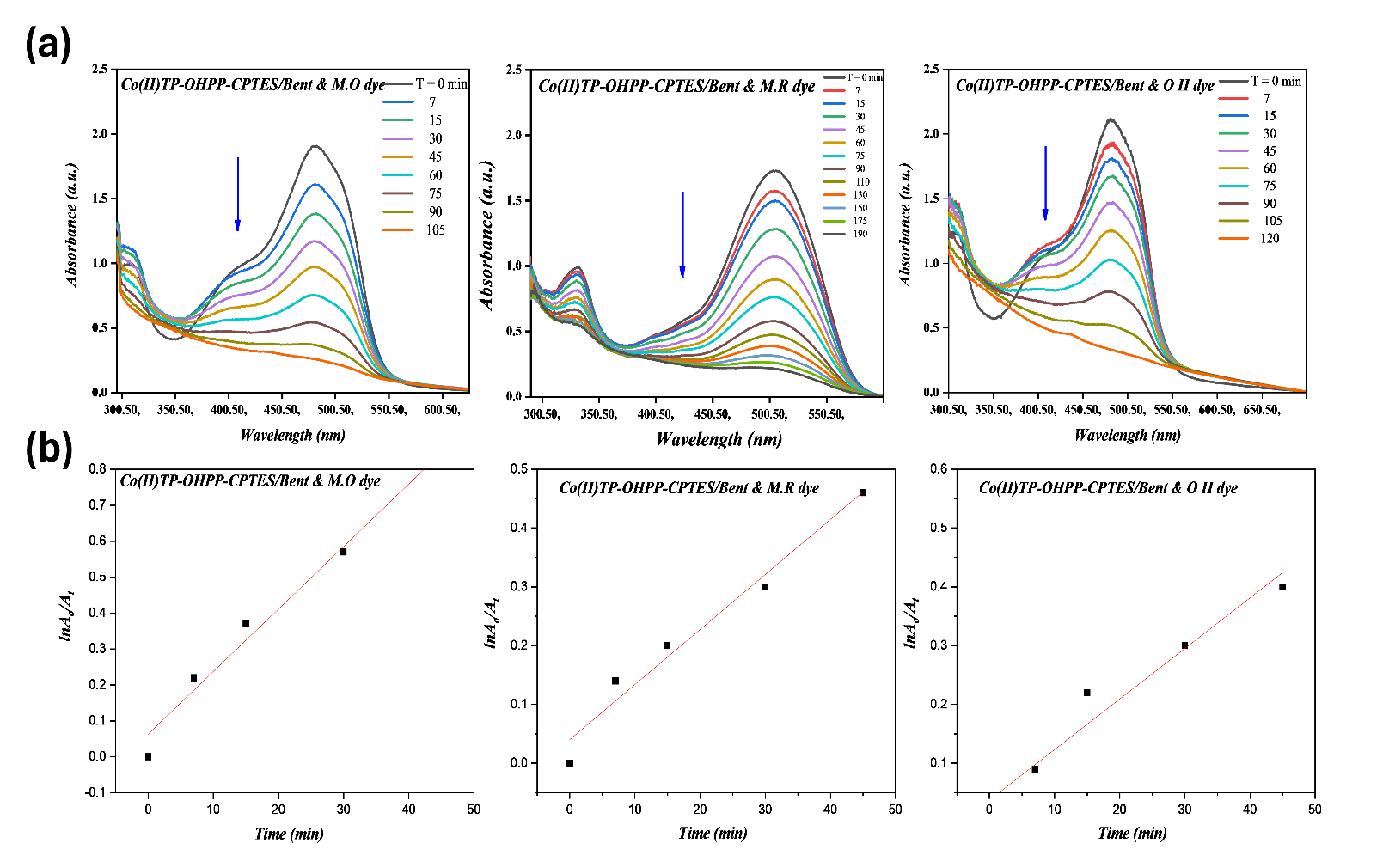


**Figure S2. (a) Electronic absorption spectra during decolorization of MO, MR and OII dyes using [Co(II)TP-OHPP]/CPTES-bent 2 composite (b) First-order plot for the degradation of MO, MR and OII with (k_obs_ = 0.011, 0.006 and 0.008 respectively). For reaction conditions: (1.33×10^−4^ M) M.O dye, (1.11×10^−4^ M) M.R dye, (1.42×10^−4^ M) OII dye, H_2_O_2_ (8×10^-2^M) in the presence of (15×10^-3^g/ml) of [Co(II)TP-OHPP]/CPTES-bent 2, pH =9 at 40 °C.**


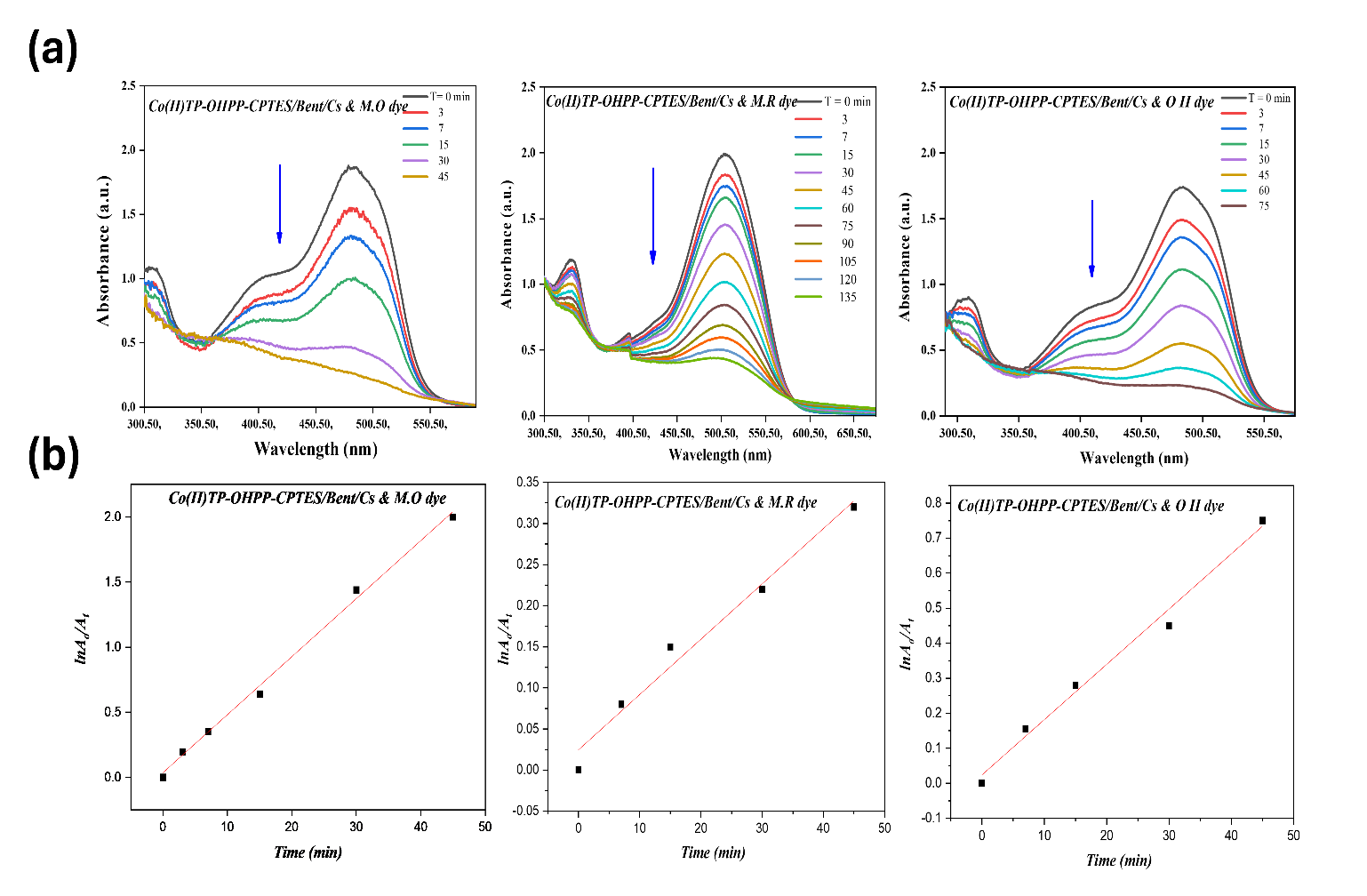


**Figure S3. (a) Electronic absorption spectra during decolorization of MO, MR and OII dyes using [Co(II)TP-OHPP]/CPTES-bent/Cs 3 composite (b)** **First-order plot for the degradation of MO, MR and OII with (k_obs_ = 0.051, 0.023 and 0.031 respectively). For reaction conditions: (1.33×10^−4^ M) M.O dye, (1.11×10^−4^ M) M.R dye, (1.42×10^−4^ M) OII dye, H_2_O_2_ (8×10^-2^M) in the presence of (15×10^-3^g/ml) of [Co(II)TP-OHPP]/CPTES-bent 2, pH =9 at 40 °C.**
